# Comparative genomic and clinicopathological analysis uncovers contrasting molecular profiles of canine and human thyroid carcinomas

**DOI:** 10.1038/s42003-025-09225-y

**Published:** 2025-12-06

**Authors:** Sunetra Das, Samantha N. Schlemmer, Rupa Idate, Susan E. Lana, Daniel P. Regan, Douglas H. Thamm, Dawn L. Duval

**Affiliations:** 1 Flint Animal Cancer Center, College of Veterinary Medicine and Biomedical Sciences, Colorado State University, Fort Collins, CO USA; 2 Department of Clinical Sciences, College of Veterinary Medicine and Biomedical Sciences, Colorado State University, Fort Collins, CO USA; 3 Department of Pathology, College of Veterinary Medicine, University of Georgia, Athens, GA USA; 4 University of Colorado Cancer Center, University of Colorado Anschutz Medical Campus, Aurora, CO USA; 5 Department of Microbiology, Immunology, and Pathology, College of Veterinary Medicine and Biomedical Sciences, Colorado State University, Fort Collins, CO USA

**Keywords:** Thyroid cancer, Thyroid cancer, Cancer genomics

## Abstract

Thyroid tumors represent 1–4% of cancers in both dogs and humans. Most canine tumors are follicular (FTC) or medullary carcinomas (MTC), unlike humans, where only 10–15% are FTC and 2% are MTC, with BRAF/NRAS or RET mutations, respectively. Here, we conduct histological and molecular analyses of canine thyroid tumors. Transcriptionally, elevated *ERBB2* expression characterizes FTC tumors, whereas MTC tumors show upregulated *RET* signaling. Elevated HER2 protein-staining and larger tumor size associate with shorter progression-free survival. Recurrent mutations are rarely observed with potential driver variants in *MEN1* (10%), *KRAS* (7%), and *TSHR* (3%), among others. Notably, mutations in DNA repair pathway genes are the most consistently shared across tumors, occurring in 60% of cases. Thus, the genomic profile of canine FTC differs significantly from that of humans, with limited reliance on RAS/RAF signaling for oncogenic progression. Conversely, RET signaling likely underlies tumorigenesis in both canine and human MTC.

## Abstract (web summary)

Comprehensive molecular analysis of canine thyroid cancer (TC) uncovers distinct oncogenic pathways: ERBB2/HER2 activation in follicular TC, and RET signaling in medullary TC, mirroring human TC and highlighting species-specific mechanisms.

## Introduction

Thyroid carcinomas (TC) are endocrine tumors that arise from either thyroid follicular cells (thyrocytes, which produce thyroglobulin, thyroxin, and colloid) or medullary/parafollicular cells (C-cells, which produce calcitonin). Thyroid cancer accounts for 1–4% and 2.2% of all cancer types in dogs and humans, respectively^1^, https://seer.cancer.gov/statfacts/html/thyro.html, and represents the most common type of endocrine cancer in both species^2^. Canine TC can be categorized into two types: follicular TC (FTC) and medullary TC (MTC), accounting for approximately 63.6–87.5% and 12.5–36.4% of cases, respectively^3,4^, with rarely reported poorly differentiated/anaplastic TC. FTCs can be further classified by histologic subtype as follicular (11.9–32.0%), follicular-compact (32.0–58.0%), papillary (3.7–7.1%), or compact/solid (13.6–40.5%) based on the World Health Organization (WHO) scheme^4–8^, the latter of which can be difficult to distinguish from MTC necessitating immunohistochemistry (i.e., thyroglobulin for FTC and calcitonin for MTC)^2,6,8–10^. In humans, the papillary subtype (PTC) is most common (84–90%), followed by FTC and MTC (~4% each) and anaplastic TC (1%)^11^.

The molecular landscapes for several subtypes of human TC have been previously reported^12–16^, while few targeted genetic studies have been reported in dogs.^17–19^ In humans, the most common cancer drivers in PTC are *BRAF* (48%), *NRAS* (7%), and *HRAS* (3%), whereas activating mutations in *RET* or *RAS* drive MTC^12,20^. *RET* fusions have been identified in up to 30% of sporadic PTC and *NTRK* gene fusions in 2–3% of cases^21^. Anaplastic TC have recurrent mutations in *TP53* (58%) and *TERT* (54%)^14,22^. In broad terms, human TC have been categorized as BRAF-like or RAS-like with the BRAF-like tumors exhibiting high levels of MAP kinase pathway activation and typically a papillary pattern, while the RAS-like tumors commonly have a follicular pattern with both MAP kinase and PI3 kinase pathway activation^12^. Although activating mutations in *KRAS* have been identified in a small number of canine TCs^17^, the remainder of these drivers of human TC have not been identified in canine studies to date.

Currently, the clinical treatment of dogs with TC is not defined by cell origin (i.e., FTC vs MTC). The preferred first-line treatment for canine TC is surgical excision, if feasible, with median survival times of greater than 36 months reported in dogs with non-metastatic, well-encapsulated tumors^23–25^. Radiation and adjunctive chemotherapy and/or immunotherapy have also been used with varying clinical benefit in certain patient cohorts^5,26–28^. Clinical findings associated with worse prognosis include tumor volume greater than 20 cm^3^, bilateral tumors, and presence of tumor vascular invasion. Up to 38% of dogs have metastasis at the time of diagnosis^26^, and these dogs may experience shorter survival^29^. The five-year survival rate of human patients with either MTC, FTC, or PTC is relatively good, 90 to 99%, (https://seer.cancer.gov/statfacts/html/thyro.html); however, anaplastic TC is highly aggressive with a one-year survival rate of 35%^30^. Molecularly targeted agents, including multi-targeted tyrosine kinase inhibitors or RET, TRK, or ALK kinase inhibitors in fusion-positive tumors, have also been utilized in human TC^31^. In contrast, molecular characterization is currently lacking to aid in the diagnosis, prognosis, and targeted treatment of canine TC.

This study documents a comparative analysis of the genomes and transcriptomes of canine and human TC. Pathological exploration of 60 canine TC tumors identified 54 FTC and 6 MTC, of which 30 tumors (25 FTC, 5 MTC) were sequenced via next-generation sequencing (NGS) technologies. In accordance with previous studies, larger tumor size at diagnosis correlated with shorter progression free intervals (PFI), although many dogs in this cohort demonstrated prolonged survival (>60 months). The mutational profiles revealed limited homology to human thyroid tumors. Transcriptomic analysis identified activation of *RET* signaling in MTC and *ERBB2* signaling in FTC tumors, with increased HER2 gene and protein expression associated with shorter PFI. The mutational landscape of canine TC shows a heterogenous population of somatic variants and, unlike human TC, lacks recurrently mutated driver genes. The commonality across these tumors lay in uncovering DNA repair pathway genes mutated in 60% of the tumors analyzed.

## Results

### Clinicopathologic data

Sixty dogs were included in this study, of which survival data were available in 53 cases and sequencing data (WES and RNAseq, *n* = 27, or RNAseq only, *n* = 3) in 30 cases (Supplementary Data 1). Clinical and histopathologic data for these animals are summarized in Tables 1 and 2, respectively. This cohort included 90% FTC and 10% MTC based on calcitonin expression. A single MTC (TC1020) had very faint calcitonin immunoreactivity on IHC, which was initially characterized as “negative” when reviewed by blinded pathologists but was later determined to be MTC based on high calcitonin transcript expression.

**Table 1:** Clinical data from 60 canine thyroid carcinomas (TC), which are also separated into putative follicular and medullary tumors (FTC and MTC, respectively) based on calcitonin expression (see Table 2) for comparisons based on tumor cell origin

	TC, n = 60	FTC, n = 54	MTC, n = 6	FTC vs MTC^a^
Age (y)				
Median (range)	10.21 (5.38–15.49)	10.39 (5.38–15.49)	8.78 (7.62–11.09)	*p* = 0.138
Breed				
Australian heeler	1 (1.7%)	1 (1.9%)	–	*p* = 0.536
Beagle	6 (10.1%)	5 (9.3%)	1 (16.7%)	
Border collie	2 (3.3%)	2 (3.7%)	–	
Dachshund	2 (3.3%)	2 (3.7%)	–	
English shepherd	1 (1.7%)	1 (1.9%)	–	
German shepherd	2 (3.3%)	2 (3.7%)	–	
German shorthaired pointer	3 (5.0%)	3 (5.6%)	–	
Golden retriever	6 (10.0%)	5 (9.3%)	1 (16.7%)	
Husky	1 (1.7%)	1 (1.9%)	–	
Labrador retriever	12 (20.0%)	11 (20.4%)	1 (16.7%)	
Malamute	1 (1.7%)	1 (1.9%)	–	
Miniature schnauzer	1 (1.7%)	1 (1.9%)	–	
Mixed breed	16 (26.7%)	14 (25.9%)	2 (33.3%)	
Portuguese water dog	1 (1.7%)	1 (1.9%)	–	
Rottweiler	1 (1.7%)	–	1 (16.7%)	
Shetland sheepdog	1 (1.7%)	1 (1.9%)	–	
Weimaraner	1 (1.7%)	1 (1.9%)	–	
Whippet	1 (1.7%)	1 (1.9%)	–	
Wirehaired pointing griffon	1 (1.7%)	1(1.9%)	–	
Sex				
Intact Female	1 (1.7%)	–	1 (16.7%)	*p* = 0.194
Spayed Female	29 (48.3%)	25 (46.3%)	4 (66.7%)	
Intact Male	4 (6.7%)	4 (7.4%)	–	
Neutered Male	26 (43.3%)	25 (46.3%)	1 (16.7%)	
Weight (kg)				
Median (range)	27.30 (7.5–57.0)	25.95 (7.5–57.0)	31.10 (16.38–36.50)	*p* = 0.218
Tumor localization				
Right	21 (35.0%)	17 (31.5%)	4 (66.7%)	*p* = 0.086
Left	21 (35.0%)	19 (35.2%)	2 (33.3%)	
Bilateral	10 (16.7%)	10 (18.5%)	–	
Ectopic	6 (10.0%)	6 (11.1%)	–	
Unavailable	2 (3.3%)	2 (3.7%)	–	
Tumor diameter (largest; cm)				
Median (range)	4.25 (2.5–17.0)	4.25 (2.5–17.0)	4.95 (3.0–8.5)	*p* = 0.717
Thyroid hormone status				
Hypothyroid	11 (18.3%)	10 (18.5%)	1 (16.7%)	*p* = 0.705
Euthyroid	24 (40.0%)	21 (38.9%)	3 (50.0%)	
Hyperthyroid	8 (13.3%)	8 (14.8%)	–	
Unavailable	17 (28.3%)	15 (27.8%)	2 (33.3%)	
Metastasis at diagnosis				
Absent	46 (76.7%)	43 (79.6%)	3 (50.0%)	*p* = 0.605
Present	13 (21.7%)	10 (18.5%)	3 (50.0%)	
Unknown	1 (1.6%)	1 (1.9%)	–	
PFI (d)				
Median (range)	1837 (126–1892)	1837 (126–1892)	ND (38–1176)	*p* = 0.253
ST (d)				
Median (range)	1892 (0–3138)	1892 (0–3138)	ND (38–1176)	*p* = 0.669

^a^Mann–Whitney U test for all parameters but progression/survival times, which were determined by Kaplan–Meier curve and log-rank test.

*p < 0.05 considered statistically significant.

*PFI* progression free interval, *ST* survival time, *ND* not defined.

**Table 2:** Histopathological data from 60 canine thyroid carcinomas (TC), which are also separated into putative follicular and medullary tumors (FTC and MTC, respectively) based on calcitonin expression (IHC and/or transcriptomic) for comparisons based on tumor cell origin

	TC, n = 60	FTC, n = 54	MTC, n = 6	FTC vs MTC^a^
Histologic pattern				
Follicular	24 (40.0%)	24 (44.4%)	–	*p* = 0.0015*
Follicular-compact	26 (43.3%)	24 (44.4%)	2 (33.3%)	
Compact	10 (16.7%)	6 (11.1%)	4 (66.7%)	
Tumor differentiation				
Well	20 (33.3%)	20 (37.0%)	–	*p* = 0.0015*
Moderate	32 (53.3%)	30 (55.6%)	2 (33.3%)	
Poor	8 (13.3%)	4 (7.4%)	4 (66.7%)	
Nuclear atypia				
Mild	34 (56.7%)	33 (61.1%)	1 (16.7%)	*p* = 0.062
Moderate	21 (35.0%)	17 (31.5%)	4 (66.7%)	
Marked	5 (8.3%)	4 (7.4%)	1 (16.7%)	
Invasion/infiltration (microscopic)				
Absent	–	–	–	*p* > 0.999
Present	60 (100%)	54 (100%)	6 (100%)	
Tumor emboli (microscopic)				
Absent	28 (46.7%)	25 (46.3%)	3 (50.0%)	*p* > 0.999
Present	32 (53.3%)	29 (53.7%)	3 (50.0%)	
Necrosis (microscopic)				
Absent	21 (35.0%)	17 (31.5%)	4 (66.7%)	*p* = 0.171
Present	39 (65.0%)	37 (68.5%)	2 (33.3%)	
Hemorrhage (microscopic)				
Absent	14 (23.0%)	11 (20.4%)	2 (33.3%)	*p* = 0.602
Present	47 (77.0%)	43 (79.6%)	4 (66.7%)	
Mineral/bone (microscopic)				
Absent	49 (81.7%)	44 (81.5%)	6 (100%)	*p* = 0.577
Present	11 (18.3%)	10 (18.5%)	–	
Calcitonin IHC				
Negative	55 (91.7%)	54 (100%)	1 (16.7%)	*p* < 0.0001*
Positive	5 (8.3%)	–	5 (83.3%)	
HER2 IHC Score (ASCO-CAP)^34,35^				
0, negative	13 (21.7%)	8 (14.8%)	5 (83.3%)	*p* = 0.0007*
1, negative	21 (35.0%)	20 (37.0%)	1 (16.7%)	
2, equivocal	15 (25.0%)	15 (27.8%)	–	
3, positive	11 (18.3%)	11 (20.4%)	–	
HER2 IHC Score (Peña)^36^				
0, negative	13 (21.7%)	8 (14.8%)	5 (83.3%)	*p* = 0.0007*
1, negative	21 (35.0%)	20 (37.0%)	1 (16.7%)	
2, equivocal	19 (31.7%)	19 (35.2%)	–	
3, positive	7 (11.7%)	7 (13.0%)	–	

^a^Mann–Whitney U test for all parameters.

*p < 0.05 considered statistically significant.

Clinical data were not statistically different between FTC and MTC (Table 1). Thyroid tumors were predominantly unilateral (70%) without a lobe predilection, although no MTC were present in bilateral nor ectopic locations. Approximately 15% of FTC were functional indicated by an increased serum total T4. No dogs with MTC were documented as hyperthyroid but thyroid hormone testing was unavailable in 2 cases. Affected dogs were typically medium to large breeds, over 7 years old, with no sex predilection. The Labrador retriever was the most frequently reported pure breed (*n* = 12), with beagles and golden retrievers also commonly affected with FTC. At diagnosis, 21% of dogs with TC had metastasis. Although MTC had numerically increased metastasis at diagnosis, the incidence was not significantly different compared to FTC. FTC metastases were found in regional lymph nodes (*n* = 8) and lung (*n* = 2), while all MTC metastases were noted in regional lymph nodes (*n* = 3).

Tumor histologic pattern and subjective tumor differentiation were statistically different between FTC and MTC (Mann-Whitney, *p* = 0.0015), with MTC tumors frequently displaying compact/solid pattern and poor differentiation. FTCs were most commonly follicular or follicular-compact (both 44.4%) patterns, with rare tumors displaying compact (11.1%) arrangements. Other routine histologic assessments (subjective nuclear atypia and the presence of microscopic tumor emboli, necrosis, hemorrhage, or mineral/bone) were not statistically different between FTC and MTC; although, no MTC contained mineral/bone. Microscopic invasion was noted in 100% and tumor emboli were appreciated histologically in 53% of all TC in this cohort, and many tumors contained hemorrhage and necrosis.

### RNASeq and whole exome sequencing analysis

#### Transcriptomic analysis identifies two discrete clusters of canine TC

**Fig. 1:**
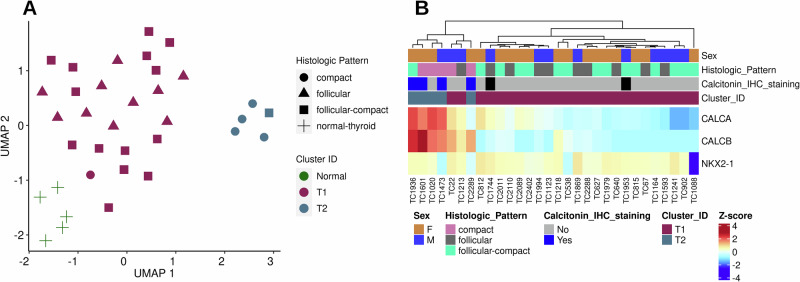
Defining clusters of canine thyroid carcinoma. **A** A set of 1013 genes with highly variable expression levels across thyroid tumor and normal samples were used for UMAP clustering. Each point represents an individual sample positioned in a two-dimensional space according to its transcriptomic profile. Samples are colored by their cluster ID and shaped by the histologic pattern defined by the pathologist, illustrating concordance between molecular clustering and histopathologic classification. **B** Heatmap of thyroid cell type markers: calcitonin A and B (*CALCA* and *CALCB)* and *NKX2-1* transcripts. The samples with high calcitonin transcript levels also had positive calcitonin IHC staining with exception of TC1020 sample. The loss of thyroid-related transcription factor (NKX2-1) transcript was observed in TC1088 sample.

The Uniform Manifold Approximation and Projection (UMAP)^32^ analysis of 30 canine TC and 5 normal canine thyroid tissues showed two distinct clusters of TC (T1/FTC, *n* = 25 and T2/MTC, *n* = 5) and one cluster of normal samples (Fig. 1A). Most follicular and follicular-compact (96%) pattern tumors were grouped in the T1/FTC cluster, except for TC22 (compact). The other 4 compact tumors were grouped in the T2/MTC cluster along with a single follicular-compact tumor (TC1936) (Fig. 1B). The T2/MTC cluster samples demonstrated high calcitonin expression at both the transcript and protein levels (Fig. 1B, Table 2). Hierarchical clustering based on gene expression of *CALCA*, *CALCB* and a key thyroid-specific transcription factor (*NKX2-1/TTF-1)* grouped the 4 calcitonin IHC-positive tumors along with histologically compact tumors and the remaining tumors with moderate to low expression of these three markers were clustered together, except for TC1088 which lacked *NKX2-1* expression.

#### Thyroid differentiation score correlates with tumor pathology and signaling genes

**Fig. 2:**
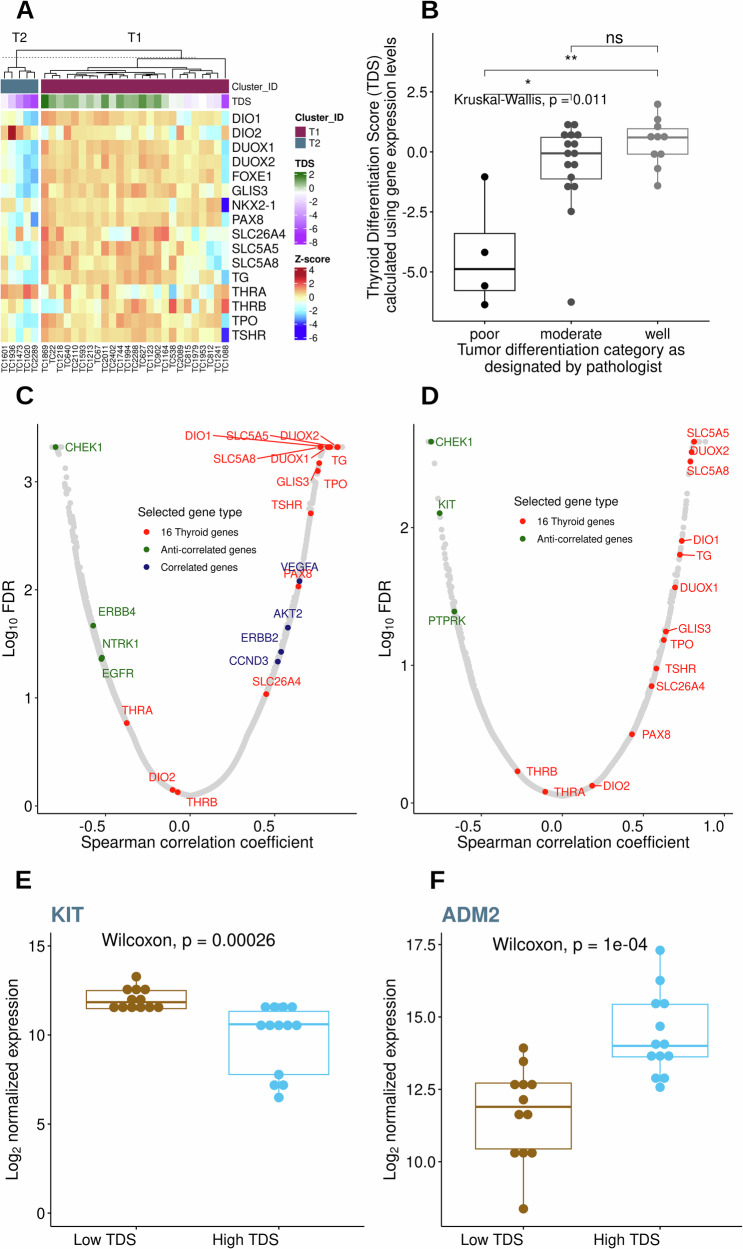
Thyroid differentiation score (TDS) and its association with tumor grade and gene expression. **A** Heatmap of 16 thyroid metabolism and function genes used in calculating the TDS. The samples were sorted in ascending order of TDS values. The two groups T1 and T2 represent FTC and MTC tumors, respectively. **B** Boxplot showing the association of tumor differentiation grades [poor (*n* = 4), moderate (*n* = 16), well (*n* = 10)] and TDS. Transcript expression levels correlated with TDS across all 30 samples (**C**) and 25 FTC samples (**D**). The Spearman correlation coefficients were plotted against Log_10_ False Discovery Rate (FDR). The red circles emphasize the 16 thyroid-related genes, and the selected positively- and negatively- or anti-correlated genes are blue and green circles, respectively. **E**, **F** Boxplot of expression data where higher transcript levels were associated with high TDS (*n* = 12) (*ADM2*) and low TDS (*n* = 13) (*KIT*) within the FTC cohort.

Thyroid Differentiation Score (TDS), calculated using expression data of 16 thyroid-specific metabolic and functional genes^12^, showed that low TDS was associated with MTC tumors and high TDS with FTC tumors in dogs (Fig. 2A), except TC1088 which had very low TDS due to the loss of *NKX2-1*. Furthermore, there was a significant association of low to high TDS values with “poor,” “moderate,” and “well” tumor histopathological differentiation categories (Kruskal-Wallis, *p* = 0.01, Fig. 2B).

Correlation of the global transcriptome with TDS identified signaling molecules enriched in 30 canine tumors (Fig. 2C, Supplementary Data 2). Most of the 16 thyroid-related genes, in addition to several cancer-related genes including *AKT2*, *VEGFA*, *ERBB2*, and *CCND3*, were positively correlated with TDS. Additionally, TDS anti-correlated genes included genes up-regulated in MTC samples, *ERBB4*, *EGFR*, and *NTRK1*, as well as *CHEK1*.

Repeating the TDS correlation analyses using only FTC samples (Fig. 2D, Supplementary Data 3) revealed key cancer genes that anti-correlated with TDS within the FTC cohort including *KIT* (Fig. 2E), *CHEK1*, and *PTPRK*. In contrast, a tumor angiogenic factor, *ADM2* (Adrenomedullin 2), was associated with a high TDS (Fig. 2F).

#### Distinct pathways regulate FTC and MTC

**Fig. 3:**
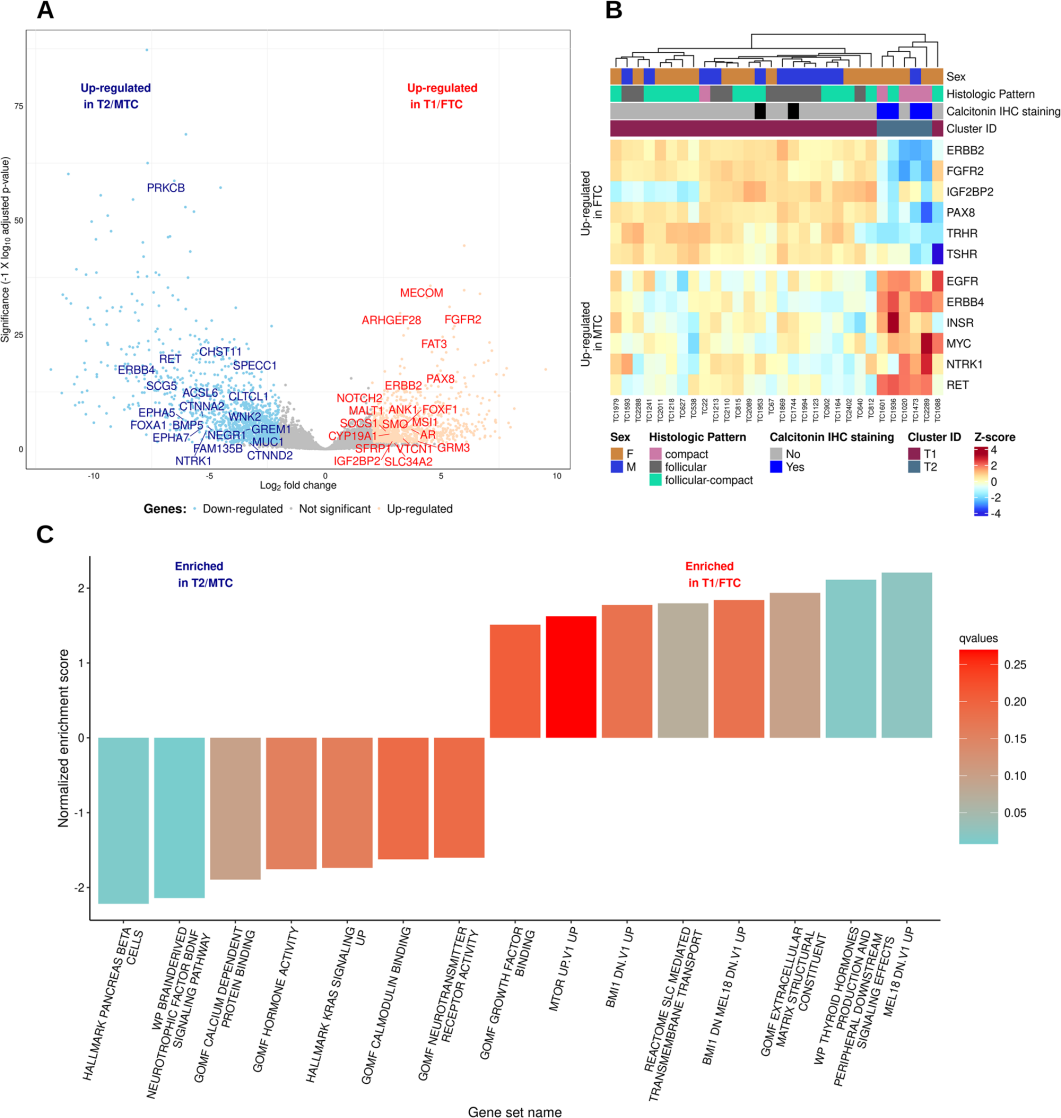
Enriched genes and pathways in canine follicular and medullary thyroid carcinomas. **A** Volcano plot of differentially expressed genes (DEGs) between clusters. Labeled genes are differentially expressed cancer genes. The downregulated (blue) and upregulated genes (red) in the volcano plot represent genes enriched in T2 cluster (MTC) and T1 (FTC) cluster, respectively. **B** Heatmap of selected DEGs representative of each cluster. **C** Significantly enriched pathways in T1 and T2 clusters. Pre-ranked differentially expressed genes were used in Gene Set Enrichment Analysis (GSEA) within the ClusterProfiler R package to identify enriched pathways. The complete list of enriched pathways is provided in Supplementary Data 4.

Differential expression analysis between the two clusters of TC identified 1348 and 1050 genes up-regulated in T1/FTC and T2/MTC groups, respectively (Fig. 3A). Some of the key cancer genes up-regulated in T1/FTC included thyroid gland-specific genes like *TRHR*, *TSHR*, *PAX8*, and fibroblast growth factor receptors, *FGFR2* and *FGFR*. Genes up-regulated in T2/MTC group included *RET*, *NTRK1*, and *MYC* (Fig. 3B). Additionally, these data showed differential expression of the ErbB family of receptor tyrosine kinases in T1/FTC and T2/MTC groups, with *ERBB2* significantly up-regulated in T1/FTC samples, and both *ERBB4* and *EGFR* genes significantly up-regulated in T2/MTC samples (Supplementary Fig. 1**)**.

The enriched gene sets/pathways identified in the T1/FTC group included extracellular matrix, growth factor binding, thyroid hormone production, and membrane-bound transporter gene sets (Fig. 3C). Oncogenic signatures related to Polycomb-group genes (BMI1 and MEL18-related gene sets) were also enriched. Gene sets enriched in the T2/MTC group included endocrine and KRAS, calcium, and neuron-related gene sets (Fig. 3C, Supplementary Data 4).

DEG analysis between canine tumor and normal thyroid samples identified 3155 up-regulated and 1230 down-regulated genes in canine FTC (Supplementary Data 5). Upregulated cancer genes included *KIT*, *DUSP4*, *ETV4*, *INSR*, and *VEGFA*. One key tyrosine kinase gene (*ERBB2*) that was also significantly upregulated in FTC compared to MTC had a log_2_ fold change of 1.53 (false discovery rate, FDR, 0.003) compared to normal thyroid samples in this analysis (Supplementary Fig. 1).

We identified 1932 and 939 up- and down-regulated genes, respectively, in canine MTC relative to normal canine thyroid tissues (Supplementary Data 6). Using the human equivalent DEG data from Minna et al.^33^, we identified a 13% gene overlap. Upregulated cancer genes in both canine and human MTC transcriptomes included *FOXA1*, *RET*, *ETV4*, and *DUSP4*. Moreover, four of the top ten DEGs identified in another study of human MTCs^15^, *CALCA, CALCB, GFRA4*, and *SEMA3E*, were also significantly upregulated in canine MTCs (Supplementary Fig. 2).

#### ERBB2 signaling in FTC

*ERBB2* was significantly over-expressed (Kruskal-Wallis, *p* = 0.0003) in FTC relative to normal thyroid and MTC (Supplementary Fig. 1). Two other EGFR family members, *ERBB4* (Kruskal-Wallis, *p* = 0.002) and *EGFR* (Kruskal-Wallis, *p* = 0.01), were significantly overexpressed in MTC compared to normals and FTC. Additionally, HER2 IHC scores, using either the ASCO/CAP^34,35^ or Peña^36^ guidelines (Supplementary Fig. 3), were statistically different between FTC and MTC (Mann-Whitney, *p* = 0.0007), with all MTC tumors considered HER2 negative (scores 0/1) while FTC spanned the entire score range (0–3). Further, *ERBB2* expression levels determined by RNAseq aligned with IHC scores (Kruskal-Wallis, *p* < 0.001, Supplementary Fig. 4).

**Fig. 4:**
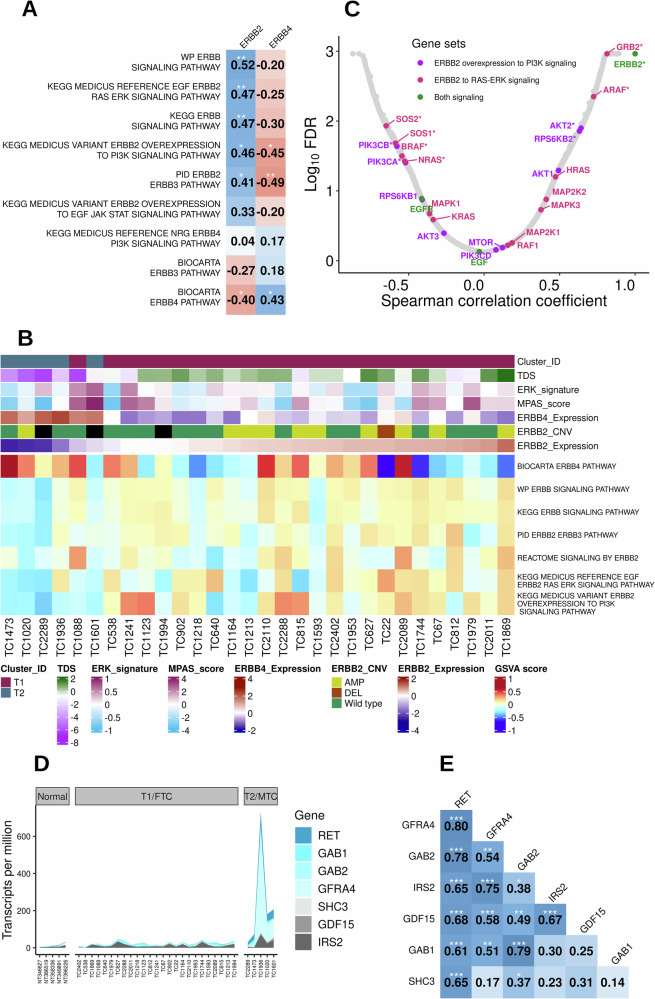
EGF receptor family and *RET* signaling genes in canine thyroid carcinomas. **A**–**C** *ERBB2* signaling in canine thyroid carcinomas. **A** Spearman correlation coefficients (*p < 0.05, **p < 0.01), of GSVA scores derived from ERBB-related gene sets and *ERBB2*/*ERBB4* expression values. **B** Heatmap of selected pathway GSVA scores and its association with *ERBB2*, *ERBB4* expression levels, *ERBB2* copy number variants (CNV) derived from Sequenza bioinformatics resource, thyroid differentiation score (TDS), ERK signature, and MPAS score. The samples were arranged in ascending order of *ERBB2* expression levels. **C** Scatterplot of Spearman coefficients and Log_10_ FDR derived from the correlation between *ERBB2* and 15,015 gene expression levels in FTC samples (*FDR < 0.05). The members of two signaling gene sets are highlighted in two different colors; purple: ERBB2 overexpression to PI3K signaling and magenta: ERBB2 to RAS-ERK signaling. The members common between the two gene sets are highlighted in green. **D**, **E** RET signaling in thyroid carcinomas. **D** Area plot displaying over-expression of seven RET signaling genes in MTC tumors. **E** The Pearson correlation matrix of genes shows that *RET* expression was significantly correlated with genes that encode ligands, adaptors, co-receptors, and activators of RET signaling pathway (*p < 0.05, **p < 0.01, **p < 0.001).

Given this differential expression pattern of EGF receptors in tumors and the presence of a HER2-driving mutation in the CTAC cell line,^37^ we assessed the enrichment of ERBB2 pathways in FTC and surveyed for *HER2*^*V659E*^ variant and HER2 protein expression. Several ERBB2-related reference pathway GSVA scores significantly correlated with *ERBB2* expression (Fig. 4A). Two additional gene sets, the ERBB2-RAS-ERK pathway and over-expression of *ERBB2* leading to PI3K signaling, were also correlated with *ERBB2* expression. *EGFR* was up-regulated in MTC samples, and three PI3K pathway genes (*PIK3CA*, *AKT2*, and *PDPK1*) were up-regulated in FTC cohort (Supplementary Fig. 5). Further, *ERBB2* expression was anti-correlated with the ERBB4 signaling pathway and vice-versa (Fig. 4A, B). When exploring gene expression correlations, ERBB2 expression was positively correlated with PI3K-AKT pathway members such as *AKT2* and *RPS6KB2*, but was inversely correlated with *PIK3CA* and *PIK3CB* expression (Fig. 4C). *ERBB2* expression was inversely correlated with 4 of the 16 members of EGF-ERBB2-RAS-ERK signaling pathway (*SOS1*, *SOS2*, *BRAF*, and *NRAS*) and positively correlated with *GRB2* and *ARAF* expression levels (Fig. 4C). While Sanger sequencing for the *HER2*^*V659E*^ variant exhibited a potential variant in six samples, no somatic variants were identified in WES or RNAseq of matching frozen samples.

#### RET signaling in MTC

Receptor tyrosine kinase, *RET* or *MEN2A/B*, gene expression was significantly up-regulated in MTC. However, no *RET* driver mutations or fusions were identified in canine MTC. Based on RET signaling in neurons^38^, we have identified a set of ligands, RET co-receptors, and downstream pathway activators that significantly correlated with *RET* transcript expression (Fig. 4D, E). Of the five known RET ligands^38^, only *GDF15* was significantly up-regulated in T2/MTC samples. GDF15 binds to GFRAL, which is a co-receptor of RET and can activate phosphorylation of ERK in the presence of wild-type RET^39^. In addition to RET and GDF15, we found that co-receptor GFRA4, an essential RET binding partner for persephin-mediated signaling, was also significantly correlated with *RET* expression^40^. Furthermore, RET signaling adaptors, *GAB1*, *GAB2,* and *IRS2*, were up-regulated in canine MTC, which in turn recruit SH2 domain-containing proteins to activate kinase signaling pathways^41^. In this study, the expression of neuron-specific gene *SHC3* (*Rai*) was correlated with *RET* expression and was significantly upregulated in the T2/MTC group compared to both the normal and T1/FTC groups (Fig. 4D).

#### Mutational spectrum and landscape

**Fig. 5:**
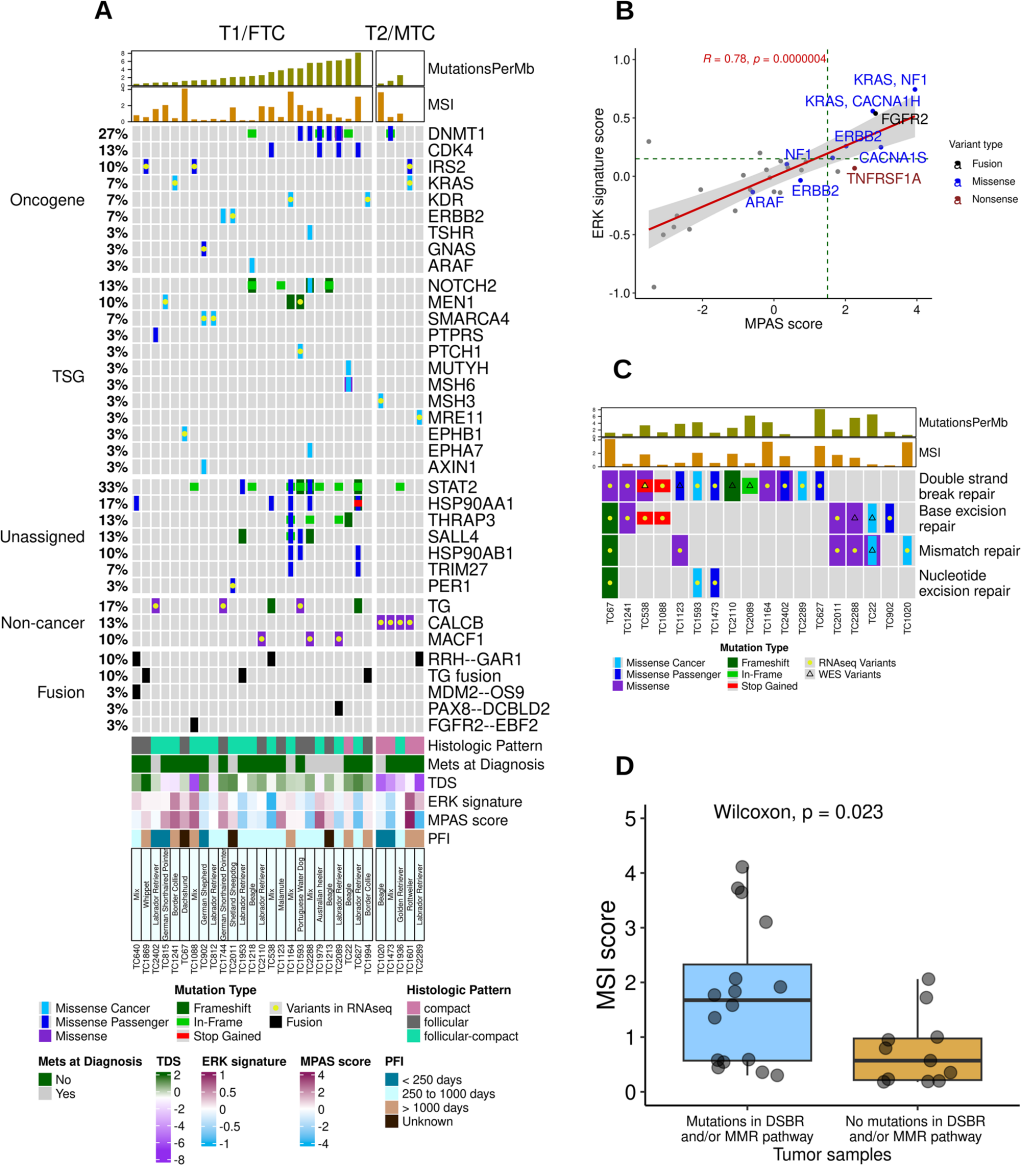
Mutational landscape of canine thyroid tumors. **A** An Oncoplot of variants identified from WES and/or RNAseq variant calling pipelines. The plot highlights cancer genes mutated in at least 10% of samples, predicted cancer-driver genes, and thyroid-related gene variants. We also integrated clinical features with the mutational landscape, alongside MPAS and ERK scores. Non-cancer genes included in the plot were thyroid-related genes (human ThyroSeq v3 genomic classifier for cancerous thyroid nodules) mutated in at least 10% of samples. **B** Scatterplot showing the MPAS and ERK score distribution across 30 tumor samples, showing a strong correlation between two pathway scores across 30 samples. Additionally, samples with mutations in genes from these two pathways were associated with high MPAS and ERK scores, Additionally, samples harboring mutations in genes from these two pathways showed elevated MPAS and ERK scores, with dotted green lines indicating the 75th percentile thresholds. **C** Illustration of samples with mutations in four different DNA repair pathways. These variants were identified either from WES and/or RNAseq pipelines. There were no significant correlations between Mutations per MB and mutations in DNA repair pathway genes. These genes are listed in Table 3. **D** Boxplot illustrating the distribution of MSI scores, grouped by samples with (*n* = 16) and without (*n* = 11) DSBR and MMR mutations. Samples with double-strand break repair (DSBR) and/or mismatch repair (MMR) gene variants exhibited a significantly higher MSI score than non-mutant cases.

Whole exome sequencing of 27 tumors and matched normals identified a somatic tumor mutational burden (TMB) of 2.9 ± 2.2 non-synonymous protein coding mutations per Mb sequenced (Fig. 5A, Supplementary Data 1). The mutational burden did not correlate with age (Pearson correlation, *p* = 0.8), weight (Pearson correlation, *p* = 0.9), tumor differentiation (Kruskal-Wallis, *p* = 0.22), or TDS (Pearson correlation, *p* = 0.09). However, dogs with low TMB had a shorter PFI (<250 days) (Kruskal-Wallis, *p* = 0.025, Supplementary Fig. 6).

Relative frequencies of DNA substitutions classified by the conventional 96 mutation types were used to construct three de novo mutational signatures from 27 TC^42^. The most common type of substitution was C > T in both FTC and MTC cohorts (Supplementary Fig. 7A). Of the three de novo mutational signatures, two were most similar to SBS5 and SBS26, respectively, and one was a novel signature (SBSA) (Supplementary Fig. 7B). Approximately 50% of tumors exhibited the SBS5 signature, while 20% harbored the SBS26 signature. Our data show a statistically significant association between SBS26 and mutational burden (Kruskal-Wallis, *p* = 0.0009, Supplementary Fig. 7C).

Cancer variants in both MTC and FTC tumors were identified using WES and RNAseq data mapped against the CanFam3.1 genome. Following variant filtering, 2587 protein-coding variants within 1581 genes were identified from WES (*n* = 27 samples) and 1930 variants in 1429 genes from RNAseq (*n* = 30) (Supplementary Data 7 and 8). The top cancer genes with recurrent variants included: *STAT2* (33%), *DMNT1* (27%), *HSP90AA1* (17%), *CDK4*, *NOTCH2*, *THRAP3*, and *SALL4* (13% each) (Fig. 5A). Variants of these genes included 24 unique SNVs, of which only one was predicted by Functional Analysis through Hidden Markov Models (FATHMM) to be a cancer driver (*NOTCH*^C1490W^), and 16 unique INDELs. The mean allelic frequency of these variants was very low, 3.3 ± 3.2%, at a median sequencing depth of 228X.

We have identified the *KRAS*^*Q155R*^ driving mutation in two samples: one FTC (TC1241) and one MTC (TC1601). Additional MAPK pathway gene variants included: *ERBB2*^*H42R & T323M*^ (TC1744 and TC2011), *ARAF*^*T235P*^ (TC1218), *NF1*^*D1430N & L928P*^ (TC22, TC1601), *TNFRSF1A*^W406^* (TC1123), two calcium channel subunit genes (TC67, TC1979), and an *FGFR2* fusion (TC1088). About 67% of these variants were associated with relatively high MAPK Pathway Activation Score (MPAS) and ERK signature scores (>75th percentile) (Fig. 5B). There was also high concordance between the MPAS score and ERK score in these samples (Pearson correlation coefficient = 0.78, *p* < 0.001). A German shepherd dog patient (TC902) also carried a *GNAS*^A204D^ variant.

The tumor suppressor gene, multiple endocrine neoplasia type 1 or *MEN1*, had three variants (A100P, N282Tfs*86, Y312Lfs*5) in canine FTC (Fig. 5A). Tumors with somatic *MEN1* mutations showed concomitant decreased expression of *MEN1* transcripts compared to samples with wild-type *MEN1* (Supplementary Fig. 8). As a tumor suppressor gene, the frameshift mutations in *MEN1* were probable drivers, and the missense mutation was a predicted cancer driver in these canine patients.

Subtype-related mutations were observed in two genes: *TG* in 20% FTC and *CALCB* in 80% MTC. Three additional canine samples carried *TG* fusions, which led to an aggregate of 32% of patients with *TG* variants. Although TG transcript levels were not significantly increased in the samples with mutations compared to wild-type *TG* samples, samples with *TG* fusions had relatively higher levels of *TG* expression (Supplementary Fig. 9A). Another FTC subtype-related variant identified was a putative driver mutation in *TSHR* (TC2288). For MTC samples, *CALCB*^A93G^ variants in 4 tumors were associated with higher expression levels of the calcitonin B gene compared to the one sample (TC2289) that lacked that mutation (Supplementary Fig. 9B).

A majority of the variants identified from mapping against Canfam3.1 were also identified using CanFam4 (Supplementary Note 1). One of the top recurrently mutated cancer genes identified from mapping to CanFam4 was *MUC4*; however, only some of these variants could be validated via Sanger sequencing due to the highly repetitive sequence of the *MUC4* gene.

#### Fusion genes

Selected gene fusions, identified from mapping RNASeq data against both CanFam3.1 and CanFam4 genomes (Supplementary Data 9), showed a heterogeneous population of fused genes that were mutated in less than 10% of the canine tumors. Putative driver fusions in canine FTC samples FGFR2--EBF2 and PAX8--DCBLD2, include gene partners *FGFR2* (VCL-FGFR2) and *PAX8* (PAX8-PPARG) that have been identified in human TC^21^. These fusions led to elevated expression of the downstream partner, but the functional implications of this are unclear. The most recurrent fusion identified was RRH-GAR1 in 3 tumors, associated with elevated expression in 2 of the 3 samples (Supplementary Fig. 10). As mentioned earlier, three samples with TG fusions were identified in FTC samples and validated using Sanger sequencing.

Potentially relevant fusions found only with the CanFam3.1 mapping and associated with elevated transcripts were MDM2-OS9 (TC640) and GNAS fusions (TC1593, TC2289, TC2402). GNAS transcripts were significantly elevated in TC2289 and TC1241 compared to the rest of the cohort.

#### Impaired DNA repair pathways

Variant calling from both WES and RNASeq data identified mutated DNA repair pathway (DRP) genes in 60% of canine TC (Fig. 5C). This included 24 genes with 26 variants that are functional components of mismatch repair (MMR), base excision repair (BER), nucleotide repair (NER), and double strand break repair (DSBR) pathways (Table 3). Comparison of tumor and matched normal WES identified 11 germline and 8 somatic DNA repair pathway mutations, while 7 variants could not be assigned as they were detected only in RNA. Mutations were observed across multiple breeds, with mixed-breed dogs accounting for most germline variants (*n* = 6); however, the limited number of cases per breed precluded identification of breed-specific predispositions (Table 3). Canine patients with DSBR and/or MMR pathway mutations (*n* = 17) had significantly higher microsatellite instability (MSI) scores compared to the rest of the cohort (*n* = 13) (Wilcoxon, *p* = 0.023, Fig. 5D). However, there was no significant association between DRP mutations and mutational burden. In addition, variants in MMR pathway genes, *MSH3*, *MSH6*, *LIG1*, *MCM9*, and *POLD3*, were associated with relatively high MSI score and/or high mutational burden in 6 tumor samples. The sample with the highest mutational burden (TC627) carried an *ATM* cancer variant, a component of the DSBR pathway.

**Table 3:** DNA repair pathway genes mutated in canine thyroid tumors

Gene Name	Sample ID	HGVSp	Sequencing Pipeline	Repair pathway	IGV validation	Genotype assignment	Breed
MSH3	TC1020	M282R	RNA	MMR	RNASeq; Tumor and Normal WES	Germline	Beagle
SIRT6	TC1241	R145H	RNA	BER, DSBR	RNASeq; Tumor and Normal WES	Germline	Border Collie
KAT5	TC67	I531L	RNA	DSBR	RNASeq; Tumor and Normal WES	Germline	Dachshund
NSD2	TC2402	A1309T	RNA	DSBR	RNASeq; Tumor and Normal WES	Germline	Labrador Retriever
MLH3	TC1123	S1103Y	RNA	MMR	RNASeq; Tumor and Normal WES	Germline	Malamute
NEIL1	TC1088	W296*	RNA	BER	RNASeq; Tumor and Normal WES	Germline	Mix
TIMELESS	TC1088	Q1283*	RNA	DSBR	RNASeq; Tumor and Normal WES	Germline	Mix
POLQ	TC538	R793*	RNA	BER, DSBR	RNASeq; Tumor and Normal WES	Germline	Mix
MDC1	TC1164	R1649Q	RNA	DSBR	RNASeq; Tumor and Normal WES	Germline	Mix
ERCC4	TC1473	H201Q	RNA	DSBR, NER	RNASeq; Tumor and Normal WES	Germline	Mix
MCM9	TC2288	R450K	RNA	MMR	RNASeq; Tumor and Normal WES	Germline	Mix
MUTYH	TC22	R658W	WES	BER, MMR	RNASeq; Tumor WES	Somatic	Beagle
MSH6	TC22	R990H	WES	MMR	Tumor WES	Somatic	Beagle
MSH6	TC22	E998D	WES	MMR	Tumor WES	Somatic	Beagle
DCLRE1C	TC2110	G207Efs*20	WES	DSBR	RNASeq; Tumor WES	Somatic	Labrador Retriever
BARD1	TC2089	Q542del	WES	DSBR	Tumor WES	Somatic	Labrador Retriever
BARD1	TC1123	L654F	WES	DSBR	Tumor WES	Somatic	Malamute
MUS81	TC538	H375N	WES	DSBR	Tumor WES	Somatic	Mix
ACD	TC2288	V266I	WES	BER	Tumor WES	Somatic	Mix
POLD3	TC67	A362Sfs*5	RNA	BER, DSBR, MMR, NER	RNASeq	Unable to assign	Dachshund
POT1	TC902	M711L	RNA	BER	RNASeq	Unable to assign	German Shepherd
RIF1	TC2402	I2399L	RNA	DSBR	RNASeq	Unable to assign	Labrador Retriever
ATM	TC627	K687Q	RNA	DSBR	RNASeq	Unable to assign	Labrador Retriever
MRE11	TC2289	L286V	RNA	DSBR	RNASeq	Unable to assign	Labrador Retriever
TP53	TC1593	I247L	RNA	DSBR, NER	RNASeq	Unable to assign	Portuguese Water Dog
LIG1	TC2011	S444R	RNA	BER, MMR	RNASeq	Unable to assign	Shetland Sheepdog

* is the standard indicator for a variant that introduces a premature stop codon.

#### Survival analyses

Many dogs in this cohort experienced prolonged survival post-thyroidectomy, with a median PFI of 1837 days and median ST of 1892 days for all TC. Similarly, for dogs with FTC, the median PFI was 1837 days and the median ST was 1892 days while the median PFI and ST were not reached for dogs with MTC due to patient censoring (Table 1, Supplementary Fig. 11A, B). The outcomes were not significantly different among dogs with FTC vs MTC.

**Fig. 6:**
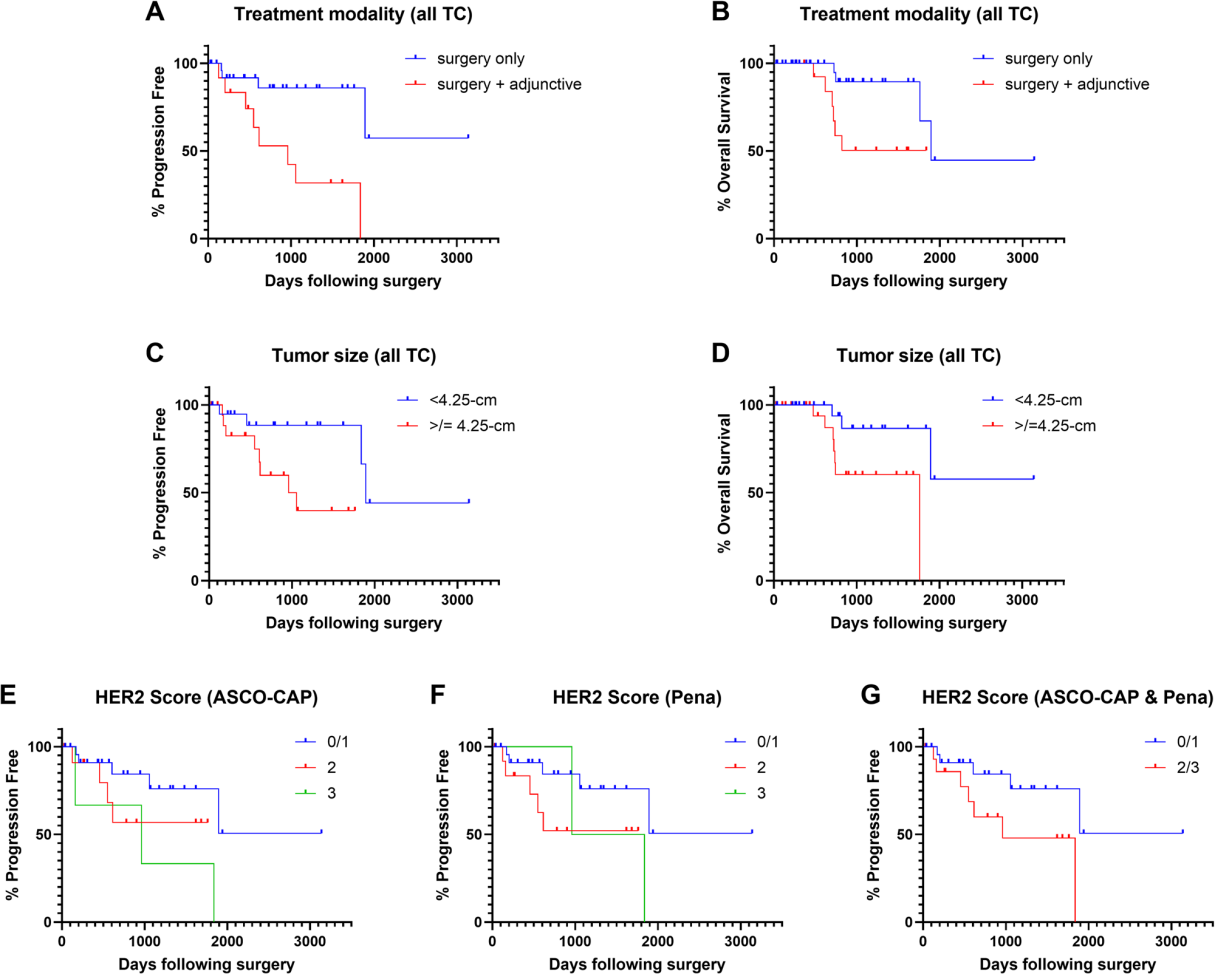
Survival analyses. **A**, **B** Treatment modality. Median PFI of dogs that received surgery only was not reached due to patient censoring, while for dogs receiving surgery and any adjunctive treatment (chemotherapy, radiation, and/or immunotherapy) was 961 days (2.63 years, *p* = 0.0024, HR 0.19, 95%CI 0.05–0.69). **C**, **D** Tumor size. Median PFI of dogs with thyroid tumors less than cohort median size (<4.25-cm) was 1892 days (5.18 years) vs 961 days (2.63 years) for dogs with tumors ≥4.25-cm; these are considered statistically different (*p* = 0.0216, HR 0.29, 95%CI 0.09–0.94). Median ST of dogs with thyroid tumors <4.25-cm was not reached due to patient censoring vs 1761 days (4.82 years) for dogs with tumors ≥4.25-cm; these are considered statistically different (*p* = 0.025, HR 0.25, 95%CI 0.07–0.92). **E**–**G** HER2 IHC score. Median PFI of dogs with ASCO/CAP HER2 Scores of 0/1 (negative) was not reached due to patient censoring, while dogs with Score 3 was 961 days (2.63 years); these were considered statistically different (*p* = 0.019, HR 0.22, 95%CI 0.02–1.90); this finding was not observed with the Pena scheme. However, when combining HER2 Scores of 2 and 3 (which was identical for ASCO/CAP and Pena schemes), median PFI was 1837 days (5.03 years) and was considered statistically different than dogs with Scores 0/1 (*p* = 0.046, HR 0.34, 95%CI 0.10–1.13).

In this cohort, 30% of dogs (*n* = 18) underwent adjunctive therapies post-thyroidectomy. Six of these dogs had metastasis at diagnosis (*n* = 13 for all dogs presenting with metastasis at diagnosis), and seven developed metastases after diagnosis. Full chemotherapy protocols were unavailable for review but included single-agent carboplatin (*n* = 10), single-agent doxorubicin (*n* = 3), combination carboplatin/doxorubicin (*n* = 2), combination melphalan/doxorubicin (*n* = 1), and combination doxorubicin/carboplatin/vinorelbine (*n* = 1). Two dogs received coarsely fractionated radiation therapy with palliative intent in addition to chemotherapy (single agent carboplatin and multiagent carboplatin/doxorubicin/vinorelbine), and one dog received toceranib (Palladia®) a tyrosine kinase inhibitor, as a single agent. Dogs that received post-thyroidectomy adjunctive therapy experienced shorter PFI and ST compared to dogs that had thyroidectomy (surgery) alone (median PFI 961 days vs median not reached due to patient censoring, *p* = 0.0024, hazard ratio (HR) 5.1, 95%CI 1.4–18.0; median ST 1892 days vs not reached due to censoring, *p* = 0.0214, HR 3.8, 95%CI 0.96–15.17; Fig. 6A, B). A similar trend was seen when thyroidectomy alone was compared to those receiving only chemotherapy as adjunctive (median PFI 1057 days vs not reached, *p* = 0.012, HR 4.28, 95%CI 1.04–17.59, median ST not reached vs 1057 days, *p* = 0.012, HR 0.26, 95%CI 0.07–0.94; Supplementary Fig. 11C, D); however, ST was not statistically different between dogs receiving surgery only and surgery plus chemotherapy (1892 days vs not reached due to patient censoring *p* = 0.099, HR 2.94, 95%CI 0.60–14.47). Metastasis at diagnosis was not considered a negative prognostic factor in our cohort (Supplementary Fig. 11E).

Of the 14 remaining clinicopathologic parameters, only two were associated with disease outcome: tumor diameter and HER2 IHC score. Increased tumor diameter was associated with decreased ST and PFI based on a median split of all TC (4.25 cm; Fig. 6C, D). Animals with smaller tumors experienced a median PFI of 1892 days compared to 961 days for animals with larger tumors (*p* = 0.0216, HR 0.29, 95%CI 0.09–0.94). The median ST of animals with smaller tumors was not reached due to patient censoring, while the median ST of animals with larger tumors was 1761 days (*p* = 0.025, HR 0.25, 95%CI 0.07–0.92). Similar findings were detected among dogs with FTC, in which dogs with tumors <4.25 cm (median split of FTC) experienced a statistically longer PFI (median 1892 days vs 961 days; *p* = 0.0217, HR 0.29, 95%CI 0.09–0.94) and ST (median not reached vs 1761 days; *p* = 0.024, HR 0.25, 95%CI 0.07–0.91) than dogs with tumors ≥4.25 cm.

HER2 IHC score was inversely correlated with PFI for all TC (Fig. 6E–G). Specifically, dogs with tumor ASCO-CAP score 3 had statistically different PFI than those with 0/1 (961 days vs not reached due to patient censoring, *p* = 0.019, HR 4.6, 95%CI 0.53–40.27). This difference was not appreciated when the Peña scheme was applied for score 3 vs 0/1 (1399 days vs not reached due to patient censoring, *p* = 0.145, HR 3.09, 95%CI 0.30–31.57). However, when scores 2 and 3 were combined (making data sets for the ASCO-CAP and Peña schemes identical), the median PFI was 961 days versus not reached for scores 0/1, which were statistically different (*p* = 0.046, HR 2.96, 95%CI 0.88–9.96). HER2 IHC scores did not have any correlations with ST.

## Discussion

We performed comprehensive characterization of a cohort of canine TC through clinicopathologic, transcriptomic, and genomic analyses, identifying distinct molecular features and prognostic indicators of FTC and MTC subtypes, and revealing similarities and differences between canine and human FTC to provide valuable insights in comparative oncology.

The distribution of FTC and MTC in our cohort aligns with previously reported incidence rates in dogs^2–4,9,10^. While calcitonin expression was generally consistent among MTC, the discordant results between IHC and transcript expression in the TC1020 specimen could be attributed to intratumor heterogeneity as different tumor tissue samples were used for IHC and genomic analyses, or less likely, rapid release and/or degradation of calcitonin by tumor cells prior to fixation^9^. Calcitonin-negative neuroendocrine TC has been infrequently encountered in the dog^43^, and an extended IHC panel to include calcitonin gene-related peptide and synaptophysin could have been considered for this specimen but was not pursued due to limited tissue remaining on the FFPE block. The clinical characteristics of affected dogs, including breed, age, and lack of sex predilection, are largely consistent with prior literature^27,44,45^. The observation that approximately 15% of FTC were functional, while a slightly lower incidence than previous reports^10,27,46^, indicates the clinical relevance of thyroid hormone production/testing in a subset of these animals. The relatively high incidence of metastasis (21% in this cohort) and large median tumor size (4.25 cm) at diagnosis likely reflect later detection, as these tumors are often found incidentally, though some animals may show clinical signs such as coughing, dysphagia, or gagging^2,47^. Furthermore, while tumor histologic pattern and subjective tumor differentiation afforded some delineation of FTC vs MTC, with MTC frequently displaying compact/solid pattern and poor differentiation, a portion of MTC were classified as follicular-compact pattern on blinded H&E review, possibly due to pseudo-follicle formation and/or entrapment of nonneoplastic follicular cells^9^, underscoring the importance of IHC to differentiate FTC from MTC^2,8^. The high rates of microscopic tumor invasion (100%) and presence of tumor emboli (53%), along with the presence of hemorrhage and necrosis, complement the large tumor sizes observed and presence of metastasis, highlighting that cases are often detected in more advanced stages.

Transcriptomic analysis effectively stratified canine TC into two distinct clusters (T1/FTC and T2/MTC), with the T2/MTC tumors displaying high calcitonin levels. High levels of serum calcitonin are used as a biomarker for MTC and other thyroid C-cell diseases in humans^48^, although a rare pathology of calcitonin-negative MTCs has also been reported^49^. The thyroid differentiation score (TDS) also distinguished FTC from MTC (with FTC displaying high TDS and MTC low TDS) and correlated with tumor differentiation (with well-differentiated tumors displaying high TDS and poorly differentiated tumors low TDS), suggesting it could be a valuable assay for canine TC subtyping. In human tumors, TDS correlates with the *BRAF*^V600E^*-RAS* score (BRS), where low and high TDS were often associated with *BRAF*^V600E^ and RAS mutations, respectively^12^. The observation of very low TDS in specimen TC1088, principally attributed to loss of *NKX2-1*, is particularly interesting as *NKX2-1* loss is correlated with de-differentiated human TC^50^, and may suggest that TC1088 might represent a de-differentiated tumor. In addition, the correlation of expression levels of signaling molecules with TDS in BRAF and RAS cohorts led to the identification of less common mutations in human TC^12^. Here, correlations between the global transcriptome and TDS also revealed distinct enriched signaling molecules in canine TC, where positive correlations with TDS were enriched with genes elevated in FTC such as *ERBB2* and *AKT2* and negative correlations identified genes elevated in MTC: *ERBB4*, *EGFR*, and *NTRK1* (Fig. 2C). Additionally, among only FTC, *ADM2* expression was associated with high TDS (Fig. 2F). ADM2 stimulates Protein Kinase A and ERK pathways in vitro, promoting cancer cell proliferation and migration. In human TC, *ADM2* is up-regulated in nutrient excess conditions, especially in the context of a high fat diet^51^. Key cancer genes negatively correlated with TDS in FTCs were *KIT*, *CHEK1*, and *PTPRK*. While the relevance of the expression of the tumor suppressor, *PTPRK*, is unclear, elevated expression of *CHEK1* was previously identified in human FTC and poorly differentiated thyroid carcinomas^52^. Higher expression of *KIT* in less-differentiated canine FTC contrasts with human PTC data, where low *KIT* expression levels were associated with low TDS often represented by less differentiated *BRAF*^*V600E*^-driven tumors^12^. As the canine tumors lack homologous *BRAF*^*V600E*^ driver mutations, the distinct *KIT* expression profile implies that it may represent an alternative mechanism to drive TC in this species compared to humans. Previously, IHC analysis of canine TC identified KIT immunoreactivity in the tumor cells of 9/15 samples^53^, but it was unclear if this expression was related to progression or reported responses to toceranib in canine TC^28^.

The differential expression analysis further revealed distinct gene sets and pathways activated in canine FTC and MTC, some of which are like human TC^15,33^. The upregulation of thyroid gland-specific genes (*TRHR*, *TSHR*, *PAX8*) and receptors (*FGFR2*, *FGFR*) in T1/FTC (vs T2/MTC) suggests their continued reliance on typical thyroid growth pathways despite malignant transformation. The enrichment of Polycomb-group gene oncogenic signatures in T1/FTC, also seen in human anaplastic TC^54^, points to potential epigenomic dysregulation in this subtype. In T2/MTC, the upregulation of *RET* and enrichment of neuron-related gene sets, mirrors key pathways in human MTC^33^. Although it is difficult to assess the specific downstream pathway of RET signaling in canine MTC from transcriptomic data alone, the relatively high expression of RET and it’s signaling components suggests activity of this pathway in MTC. In humans, overexpression of wild-type *RET* has been observed in several cancer types, including breast cancer and pancreatic ductal carcinomas, and is associated with high-grade metastatic tumors^55^. Germline mutations in human *RET* are responsible for the autosomal dominant genetic syndromes multiple endocrine neoplasia type 2 (MEN2) and familial MTC. These syndromes are characterized by an increased incidence of MTC and an increasing risk for the development of pheochromocytoma, primary hyperparathyroidism, and other endocrine neoplasms. While only approximately 25% of human MTCs are hereditary, as many as 75% of sporadic human MTC have somatic mutations in the RET oncogene^56^.

The differential expression of ErbB family receptor tyrosine kinases, with *ERBB2* significantly upregulated in T1/FTC and *ERBB4* and *EGFR* in T2/MTC, further emphasizes the distinct signaling landscapes of these subtypes. The correlation of ERBB2 with PI3K-driven signaling pathways in FTC, observed through GSVA scores and expression correlations with *AKT2* and *RPS6KB2*, aligns with previous findings in canine FTC^17^; protein expression of *ERBB2* (HER2) aligned with gene expression and distribution was also similar to a recent retrospective canine FTC study^57^. Over-expression of *ERBB2* has been attributed to genomic amplification or transcriptional regulation and is usually coupled with poor clinical outcomes in human cancers^58^. Thus, ERBB2 overexpression may have prognostic value (shorter PFI, discussed below) and serve as a potential novel therapeutic avenue for canine TC.

In our cross-species comparisons, we identified notable gene overlaps between canine and human TC transcriptomes, including *KIT*, *DUSP4*, *ETV4*, *INSR*, and *VEGFA* in FTC and *FOXA1*, *RET*, *ETV4*, *DUSP4, CALCA, CALCB, GFRA4*, and *SEMA3E* in MTC. These shared molecular signatures highlight conserved pathways in TC carcinogenesis, while also highlighting unique species-specific aspects. For instance, the *FOXA1* gene was ubiquitously over-expressed in human MTC, but not in FTC, and might serve as a diagnostic marker^59^.

Investigating the mutational landscape, the somatic TMB in canine TC was similar to human PTC TMB^12^ but higher than other canine cancers (e.g., osteosarcoma, hemangiosarcoma and melanoma, where median TMB was ≥ 1 mutations per Mb)^60^, suggesting a moderate level of genomic instability. However, unlike human cases, the lack of correlation between TMB and age or tumor differentiation in dogs highlights species-specific differences in factors influencing mutational burden. The association of low TMB with shorter PFI is an intriguing finding that warrants further exploration. The identification of mutational signatures similar to SBS5 and SBS26 indicates some conserved mutational processes between human and canine TC^42^. The etiology of SBS5 is unknown but correlates with age and has been observed in human bladder cancers with mutation of the ERCC2 nucleotide excision repair enzyme and cancers due to tobacco smoking^61^. The SBS26 signature is associated with defective DNA mismatch repair^62^. The significant association between SBS26 and mutational burden suggests impaired DNA mismatch repair may contribute to higher mutation rates in some canine TC. The diverse and heterogeneous mutational landscape observed in canine TC, with top variants in genes like *STAT2, DMNT1*, and *HSP90AA1*, distinctly contrasts with common recurrent driver mutations (*BRAF, RAS*) seen in humans^12^. The identification of *KRAS*^*Q155R*^ as a driver mutation in both FTC and MTC samples, along with other MAPK pathway variants as indicated in Fig. 5B, indicates that while BRAF is not a dominant driver as in humans, the MAPK pathway remains a relevant target for potential therapeutic strategies in canine TC. The presence of probable driver mutations in the tumor suppressor gene *MEN1* in 10% of canine TC, accompanied by decreased *MEN1* transcript expression, suggests a role for this pathway in canine FTC, mirroring its involvement in various human endocrine tumors^63^. The *GNAS*^A204D^ variant, while previously identified as a likely novel^19^, being predicted as a cancer passenger mutation in this cohort suggests this alteration might not be a cancer driver in canine TC. Hotspot activating mutations (R201 and Q227) and amplifications of GNAS have been identified in human tumor types including pituitary, pancreatic, colorectal, and TC^64^. The heterogeneity in the tumor mutational landscape is distinctly different from the human TC counterpart; however, similar genetic heterogeneity has been seen in human neuroendocrine tumors^65^.

In addition to short variants, our analysis of fusion genes revealed a few putative driver fusions like FGFR2--EBF2 and PAX8--DCBLD2 in FTC, with gene partners homologous to those in human TC fusions^21^, suggesting their functional relevance in canine TC. The most recurrent fusion, RRH-GAR1, is of unknown functional significance and warrants further investigation given the elevated expression of both fusion partners in tumors bearing this fusion. The absence of recurrent RET fusions, common in human TC^66^, reinforces the distinct genomic landscape of canine TC, aligning with our finding of RET overexpression without typical fusion. The presence of TG fusions further highlights the uniqueness of canine FTC. Widespread genomic instability is also suggested by the detection of mutated DNA repair pathway genes in 60% of canine TC, including MMR, BER, NER, and DSBR pathways. The strong association between DSBR/MMR mutations and significantly higher MSI scores directly implicates these impaired pathways in promoting microsatellite instability in canine TC. While some DRP variants may be germline, their contribution to genomic instability, exemplified by the *ATM* cancer variant in the highest mutational burden sample (TC627), could explain the heterogeneous mutational landscape and facilitate tumor progression in canine TC.

Finally, survival analysis revealed prolonged median PFI and ST for the overall cohort, with no significant differences between FTC and MTC outcomes, consistent with what has been previously reported^10^; however, the MTC cohort in this study was underpowered and future studies are needed to clarify the clinical distinction of FTC vs MTC. Metastasis at diagnosis was also not considered a negative prognostic factor in our cohort, which is supported and conflicting with other studies^29,67^; as such, the significance of this finding in canine TC remains unclear. The finding that dogs receiving adjunctive therapies post-thyroidectomy experienced shorter PFI and ST compared to surgery alone is a complex observation and likely reflects the clinician’s decision to recommend additional treatment for dogs with perceived poor prognoses (e.g., those with metastasis at diagnosis or aggressive tumor features), rather than a negative effect of the therapies themselves. This is not an uncommon challenge in retrospective studies and makes direct causation difficult to establish^67^, particularly as there is no clearly demonstrated clinical benefit of adjunctive therapy post-operatively in canine TC^29,68^. The association of increased tumor diameter with decreased outcomes (where smaller tumors correlated with significantly longer survival) is a critical prognostic finding, consistent with some but not all prior canine TC literature^10,23,29,44,67,69^. This finding underscores the importance of early detection in improving patient outcomes, as smaller tumors may be more amenable to complete excision and earlier excision could prevent or delay metastasis, as well as other complications from tumor growth and invasion. The inverse correlation between HER2 IHC score and PFI (where higher score, particularly ASCO-CAP score 3 or combined 2 + 3) suggests HER2 may serve as a prognostic and potential therapeutic biomarker in canine TC. As mentioned previously, over-expression of *ERBB2* is associated with poor clinical outcomes in human cancers^58^. In canine mammary carcinomas, HER2 overexpression (IHC scores 3 ± 2) has been correlated with longer PFI and ST but also with presence of higher histologic grade and even proliferative index, although these are inconsistently associated in the literature^70–75^. As such, the diagnostic and prognostic utility of HER2 IHC remains to be clarified in the veterinary literature and future directed studies are needed.

In all, this study demonstrates notable differences in the gene-expression and mutational landscapes of human and canine TC. While canine MTC may be similarly driven by RET signaling (although, unlike humans, no driver mutations nor fusions were detected in our dataset), canine FTC do not appear to rely on RAS/RAF signaling and display a heterogenous mutational landscape, employing a variety of processes for oncogenesis (including ERBB2 and PI3K, as well as dysregulation of epigenomic and DNA repair pathways). Notably, the EGFR/ERBB family displayed differential expression among canine TC, with FTC demonstrating upregulated expression of ERBB2 and MTC upregulated expression of ERBB4. ERBB2 transcriptomic expression correlated with HER2 protein expression in FTC and increased protein expression correlated with decreased PFI, raising the possible utility of HER2 as a diagnostic, prognostic, and therapeutic marker for canine FTC.

Overall, canine TC appears slowly progressive with a median PFI of around 5 years and median survival time of 5.2 years after diagnosis/surgery in this cohort. While survival metrics comparing tumor origin were not statistically different, the low number of MTC in this study may have diminished our ability to detect differences and further studies are needed. Clinical factors associated with diminished outcomes included larger tumor diameter at diagnosis and use of post-operative adjunctive treatment. Conflicting with previous studies, metastasis at diagnosis, tumor localization, and presence of tumor emboli were not associated with worse outcomes. In conclusion, integrating clinicopathologic and genomic data provides additional insights into canine TC, comparative oncology, and emphasizes the importance of larger canine studies to validate prognostic factors and inform therapeutic approaches.

## Methods

### Ethics statement

Dog samples were archived during clinical treatment by the Colorado State University (CSU) Flint Animal Cancer Center Biorepository with owner consent, and the CSU Institutional Animal Care and Use Committee approved all animal procedures performed in this study under the protocol (KP 652). We have complied with all relevant ethical regulations for animal use.

### Sample acquisition and processing

The canine thyroid tumors were acquired from the CSU Flint Animal Cancer Center Biorepository. All tumors had formalin-fixed, paraffin embedded (FFPE) tissue, and a subset also had frozen tissue sections. FFPE tumor tissue blocks were routinely processed for hematoxylin and eosin (H&E) staining and evaluated by two veterinary pathologists (SNS & DPR) to confirm adequate tumor sample for inclusion in pathologic and genomic analyses. Both DNA and RNA were extracted from each available frozen tumor sample using TRIzol (Invitrogen, Catalogue # 15596026) according to the manufacturer’s protocol. Matched normal sample DNA was also extracted for whole exome sequencing (WES; Supplementary Data 1). The DNA and RNA samples were purified using DNeasy or QiaAMP DNA Blood mini kits and RNeasy (Qiagen, Catalogue #69504, 51104, 74004), respectively. RNA/DNA purity, quantity and integrity were determined by NanoDrop (ThermoFisher Scientific) and TapeStation 4200 (Agilent, CA, USA) analysis prior to DNA and RNA-seq library preparation. The Zymo-Seq RiboFree Total RNA Library Kit was used (Zymo Research, CA, USA, Catalogue #R3000) with an input of 300 ng of total RNA to generate RNAseq libraries. Paired-end sequencing reads of 150 bp were generated on a NovaSeq 6000 (Illumina Inc., CA, USA) sequencer at a target depth of 60 million paired-end reads per sample. For WES, the Agilent SureSelect XT All Exon Canine V2 (part number: 931198, Santa Clara, CA) for Illumina Paired-End Multiplexed Sequencing Library kit was used to create genomic DNA libraries that were also sequenced on a NovaSeq6000 generating 150 bp paired-end reads. Raw sequencing reads were de-multiplexed using bcl2fastq. Sequencing and library prep for DNA and RNA was carried out at the Genomics Shared Resource, University of Colorado Anschutz Medical Campus (RRID: SCR_021984). In addition, we downloaded RNAseq fastq files of normal thyroid tissues from an open-access dog epigenomic resource, BarkBase (https://barkbase.org/, BioProject PRJNA396033)^76^.

### Clinicopathological data

Medical records were reviewed to identify clinical data including: age at diagnosis (i.e., surgery), sex (including neuter status), weight, breed, tumor localization, largest tumor diameter (cm), and thyroid hormone testing (if available). Survival data (progression free interval, PFI, and overall survival, ST; determined as time from surgery to disease progression, death due to disease, or last known follow-up) were determined from CSU medical records and/or contact with referring veterinarians.

The FFPE tumor samples were subjected to histopathologic characterization (performed on H&E-stained slides) and immunohistochemical (IHC) staining for calcitonin and HER2 by two veterinary pathologists, with consensus for each evaluated parameter. Histopathologic factors assessed were: histologic pattern (i.e., follicular, follicular-compact, and compact, based on the WHO scheme^77^); degree of differentiation (i.e., well, moderate, and poor); degree of nuclear atypia (i.e., mild, moderate, or marked); and presence of microscopic invasion/infiltration, tumor emboli, necrosis, hemorrhage, and mineral/bone. Given small tumor specimens, a single marker (calcitonin) was used to determine cell origin on IHC, as previous literature suggested a high concordance of calcitonin immunoreactivity in canine MTC^8,10^. For IHC, slides were stained on the BOND Rxm system using the BOND Polymer Refine Detection kit (Leica Biosystems Inc.) with the following steps: (1) antigen retrieval with Tris-EDTA buffer, pH 9.0, for 20 min at 100 °C (BOND Epitope Retrieval 2, Leica), (2) incubation at ambient temperature for 15 min with primary rabbit polyclonal antibodies for calcitonin (Abcam, ab8553; 1:200 dilution, validated based on immunoreactivity in canine thyroid (normal and carcinoma) tissue specimens – personal communication with Kansas State Veterinary Diagnostic Laboratory) or HER2 (Dako, A0485; 1:400 dilution, previously validated in canine cells/tissue^78^) diluted in BOND Primary Antibody Diluent (TBS), and (3) incubation with BOND Polymer at ambient temperature for 25 min, exposed to chromogen (DAB) and counterstained with hematoxylin. Tissue samples were considered calcitonin positive if the majority of tumor cells showed strong cytoplasmic reactivity. The HER2 samples were graded based on American Society of Clinical Oncology–College of American Pathologists (ASCO-CAP)^34,35^ and Peña^36^ recommendations for human and canine specimens, respectively, where grade 0–1 are negative, 2 is equivocal, and 3 is positive (Supplementary Fig. 3).

### Processing and analysis of sequencing data

The paired end RNAseq reads (range: 51.9–116.3 million) were mapped against CanFam3.1 (Ensembl version 99) and CanFam4 (UU_Cfam_GSD_1.0, NCBI RefSeq assembly: GCF_011100685.1), with STAR using -genecount parameters (v2.6.1a)^79^. The new canine genome (CanFam4) was also supplemented by three Y chromosome sequences from a Labrador retriever (ROS_Cfam_1.0, GCF_014441545.1) assembly^80^. Gene count data were normalized with DESeq2 (v1.26.0) median of ratios method^81^. Additionally, transcripts per million (TPM), a method for normalizing gene expression levels, was calculated for each gene using TPMCalculator^82^. Normalized count data were log-transformed and scaled for plotting and downstream analyses. The WES paired reads were also mapped against both CanFam3.1 and CanFam4 genomes using BWA. Prior to variant calling, the BAMs were processed in accordance with GATK best practices^83^.

### Clustering approach for sample categorization

The Uniform Manifold Approximation and Projection (UMAP) dimension reduction algorithm was used to group tumor and normal samples into distinct clusters^32^ using DEseq2 log-transformed normalized data as input. A subset of genes with mean expression log_2_  >  2 and mean variance log_2_  >  6 (*n*  =  1013) were used for clustering samples to reduce noise and focus on biologically relevant features.

### Differential expression and pathway enrichment analysis

DESeq2 was used to run differential expression (DE) analyses between the two canine thyroid cancer tumor clusters^81^. In both principal component analysis (PCA) and hierarchical clustering of the samples (Fig. 1B), TC1088 separated from the main cluster of thyroid tumors. Further inspection revealed that TC1088 was the only sample with loss of *NKX2-1*, a key thyroid transcription factor that regulates gene expression required for essential thyroid function. Accordingly, this sample was considered an outlier and excluded from differential expression analyses. The sample TC1088 was identified as an outlier and was eliminated from DE analysis. For the DEG analysis pipeline, raw gene counts were filtered to retain only genes with at least 10 reads in at least 5 samples. Following the median of ratios count normalization, DESeq was used to identify differentially expressed genes (DEGs) with log_2_ fold change >2 and adjusted *p* < 0.05. The DEGs were also identified between normal thyroid samples and each tumor cluster. Enriched pathways (FDR < 0.25) in each cluster were identified using pre-ranked Gene Set Enrichment Analysis (GSEA) through the ClusterProfiler package in R, which employs the Kolmogorov-Smirnov goodness-of-fit test^84^.

### Gene fusion identification and processing

STAR-fusion was used to identify fusion genes from CanFam3.1 and CanFam4 genome assemblies^85^. Two canine gene fusion databases were created from both genome assemblies using prep_genome_lib.pl script from STAR-fusion. Using fastq reads as input, gene fusions were identified by the STAR-fusion tool. The fusions were filtered and manually curated to identify putative drivers. We used the following criteria to eliminate potential false-positives: (a) fusion genes where both partners are from the same gene family (e.g.: PBX2-PBX3), (b) fusions without official gene names, (c) fusions identified in >80% of the samples, and (d) fusions that were identified in both TC and normal thyroid tissues. The fusion genes were further curated to identify fusions with known cancer genes (OncoKB database). Selected fusion genes were also validated via Sanger sequencing. The forward and reverse primer sequences were reported in Supplementary Table 1.

### Signaling and thyroid differentiation score

#### MPAS score

The MAPK Pathway Activation Score (MPAS) developed by Wagle et al. was used to quantify relative MAPK activity using gene expression data in thyroid tumor samples. The expression of 10 downstream MAPK pathway targets (*CCND1*, *DUSP4*, *DUSP6, EPHA2*, *EPHA4*, *ETV4*, *ETV5*, *PHLDA1*, *SPRY2*, *SPRY4*) were used to calculate the score^86^.

$${MPAS}=\frac{\,\sum {MPAS\;gene}{\ Z}-{scores}\,}{\sqrt{{\mathrm{Number}}}\; {{\mathrm{of}}}\; {{\mathrm{genes}}}}$$

#### ERK score

The ERK score or ERK output signature was developed by Pratilas et al. which distinguishes between high and low RAS/MEK pathway activation^87^. The score was calculated by computing the mean of the Z-scores for 52 genes, which were selected based on their involvement in the RAF-MEK-ERK signaling pathway. Usually, the ERK score is greater in human BRAF^V600E^ carrying tumors compared to RTK-activated tumors. In the canine dataset (CanFam3.1), 11 of the 52 genes were not annotated, hence the score was calculated using 41 genes.

$${ERK}\;{Score}=\frac{1}{n}{\sum }_{i=1}^{n}{Z}_{i}$$

- n = the number of genes (in this case, *n* = 41)
- Z = Z-score of log_2_ normalized count data

#### TDS score

TDS, or thyroid differentiation score, was calculated using the log_2_ DESeq2 normalized count of 16 thyroid-related genes^12^. For each of the 16 selected genes, we first calculated the median log_2_ expression value across all samples. The expression levels of the 16 thyroid function genes were then median-centered and summed to generate the TDS score for each sample. The TDS score thus reflects the overall expression profile of thyroid function genes, normalized for each gene’s median expression across samples.

### Somatic variant calling and downstream processing

Duplicate reads were marked with Picard tools (v1.119) and somatic variants were called using GATK Mutect2 (v4.1.2.0)^83^. Variants were called from both RNAseq and WES data. For the WES variant calling pipeline, germline variants were eliminated using three different resources: (a) matched normals, (b) a panel of normals created from 118 in-house normal tissue or PBMC samples using the GATK pipeline, and (c) an external germline resource identified from 722 dogs (~90 million population variants)^88^. The remaining variants were processed using the filterMutectCalls GATK function, and variants with a PASS notation in the FILTER column were characterized as somatic variants. For the RNAseq variant calling pipeline, variants were identified using GATK HaplotypeCaller. Germline variants were filtered out following the same approach as the WES pipeline, with the exception that a matched normal was not used. Additional filtering criteria included a read depth (DP) greater than 10, an alternate allele count greater than 5, removal of splice variants, and selection of variants with an allelic frequency between 0.25 and 0.85. The VCF files with somatic variants were converted to MAF (Mutation Annotation File) format (https://docs.gdc.cancer.gov/Data/File_Formats/MAF_Format/) using the perl code: vcf2maf.pl (https://github.com/mskcc/vcf2maf). Each variant was mapped to only one of all possible gene transcripts using the “canonical” isoform from Ensembl database (v99). The cancer-associated genes were curated using COSMIC (v94) and OncoKB databases^89^. These gene lists were then used to cross-reference with canine genes carrying variants. Orthologous protein-coding positions in human protein sequences that corresponding to canine cancer gene variants were identified using custom code (Supplementary Software 1). To achieve this, full-length human and canine protein sequences for the genes of interest were pairwise aligned using BLAST, and the resulting alignments were processed with custom R functions to determine the orthologous position of each variant amino acid in the human sequence. This procedure ensures that all positions reported as orthologous are directly supported by sequence alignment. These orthologous human variants were then analyzed using Functional Analysis through Hidden Markov Models (FATHMM) to predict cancer driver or passenger mutations^90^.

The criteria for selecting genes to generate the oncoplot were as follows:

a. Cancer-associated genes (COSMIC and OnkoKB database) with recurrent mutations in at least 10% of the samples.

b. Genes with mutations present in only one sample, provided the mutation was predicted to be a cancer driver by FATHMM.

c. Thyroid-related genes with recurrent somatic mutations. These thyroid-related genes were identified by cross-referencing canine genes with variants against the human ThyroSeq v3 gene list^91^.

The mutational signatures of the samples were explored using the MutationalPatterns R package^92^. The microsatellite instability (MSI) score for each tumor was calculated using MSIsensor-pro^93^. The mutational burden for each sample was defined as the number of protein-coding somatic variants normalized to the total number of megabases sequenced.

Allele-specific copy number variant (CNV) genes were identified using the *Sequenza* (v2.1.9999b1) tool with tumor and matched normal BAM files from whole-exome sequencing data^94^. Allele frequencies were used to calculate tumor-to-normal depth ratios, and allele-specific segmentation was generated with the copynumber R package. This approach yields allele-specific CNV profiles, providing estimates of total copy number, major and minor allele copy numbers, and tumor cellularity and ploidy.

### DNA repair pathway gene selection

Using DNA repair gene sets from the MSigDB database, we identified genes harboring variants and mapped them to their corresponding pathways. Specifically, we focused on four major DNA repair pathways: mismatch repair (MMR), base excision repair (BER), nucleotide excision repair (NER), and double-strand break repair (DSBR). The identified variants were validated by manual inspection in Integrated Genome Viewer (IGV) using BAM files from whole-exome sequencing, RNAseq, and matched normal samples. Genotypes were assigned based on variant presence across datasets: variants detected in the matched normal sample were classified as germline; those observed in WES and/or RNAseq but absent in the normal were classified as somatic. Variants detected exclusively in RNAseq could not be confidently assigned a genotype. Although these RNAseq variants were screened to exclude likely germline events, there remains low confidence in genotype assignment when relying solely on allelic frequency.

### Variant validation using Sanger sequencing

Validation of selected variants and gene fusions was conducted using Sanger dideoxy sequencing (Genewiz) of PCR amplified products from genomic DNA or cDNA. Following amplification of identified regions, the amplicons were evaluated by gel electrophoresis, gel isolated, and sequenced using either the forward or reverse amplification primers. Primers were designed from STAR-fusion predicted cDNA sequences of fusion genes or identified variants using Geneious or Primer3Plus (https://www.primer3plus.com/) and purchased from Integrated DNA Technologies (Coralville, IA, USA). The list of primer sequences was provided in Supplementary Table 1. Results were aligned with the reference sequence using Geneious (Boston, MA, USA) software to identify variants.

### Statistics and reproducibility

Statistical analyses were done using R statistical tool (v3.6.1 and v4.1.1) and GraphPad Prism software (v10.4.1). Correlations were assessed by calculating Pearson and Spearman correlation coefficients, and the FDRtool R package was used to correct for multiple testing. The oncoplots and heatmaps were plotted using ComplexHeatmap R package^95^. Bar charts and boxplots were plotted using R package ggplot2^96^. Clinicopathological data comparisons between FTC and MTC were assessed via nonparametric comparison with a Mann-Whitney U test. Survival analyses (PFI and ST) were performed using Kaplan–Meier curves with log-rank test and COXPH analyses; animals with metastasis noted at diagnosis were excluded from PFI analyses. Significance was set at *p* < 0.05.

### Reporting summary

Further information on research design is available in the Nature Portfolio Reporting Summary linked to this article.

## Supplementary information

Supplementary Information

Description of Additional Supplementary Files

Supplementary Data 1-9

Supplementary Software 1

Reporting Summary

## Data Availability

The raw fastq files for WES have been submitted to SRA database under BioProject number: PRJNA1222422. The RNAseq fastq files have been submitted to GEO database under accession number: GSE289443. Source data behind all figures are available on figshare^97^. All other data are available from the corresponding author on reasonable request.

## Code availability

The scripts and tool versions used for the WES and RNAseq pipelines are available at https://github.com/sunetrad/Canine_ThyroidCarcinoma_Omics_Pipeline.git. Custom codes, along with input and output files for cross-species amino acid position mapping, is provided in Supplementary Software 1^98^.
