# Supplementary material for: Comparative genomic and clinicopathological analysis uncovers contrasting molecular profiles of canine and human thyroid carcinomas
Source: Commun Biol. 2025 Dec 6;9:4. doi: 10.1038/s42003-025-09225-y (PMC12765017; doi:10.1038/s42003-025-09225-y)
Supplement: Supplementary file 1 — Supplementary Information [file 42003_2025_9225_MOESM1_ESM.pdf]

Supplementary Information

Comparative genomic and clinicopathological analysis uncovers contrasting molecular profiles of canine and human thyroid carcinomas

Sunetra Das[1,2¶], Samantha N. Schlemmer[1,2,3¶*], Rupa Idate[1,2], Susan E. Lana[1,2,5], Daniel P. Regan[1,4,5], Douglas H. Thamm[1,2,5], Dawn L. Duval[1,2,5]

Affiliations:

1. Flint Animal Cancer Center, College of Veterinary Medicine and Biomedical Sciences, Colorado State University, Fort Collins, Colorado, USA
2. Department of Clinical Sciences, College of Veterinary Medicine and Biomedical Sciences, Colorado State University, Fort Collins, Colorado, USA
3. Department of Pathology, College of Veterinary Medicine, University of Georgia, Athens, Georgia, USA
4. Department of Microbiology, Immunology, and Pathology, College of Veterinary Medicine and Biomedical Sciences, Colorado State University, Fort Collins, Colorado, USA
5. University of Colorado Cancer Center, University of Colorado Anschutz Medical Campus, Aurora, Colorado, USA

*Corresponding author
Email: sschlemmer@uga.edu (SNS)

¶These authors contributed equally to this work.

Table of Contents

**Supplementary File-Type #**	**Title**	**Page #**
**Figure 1**	Normalized expression data of selected differentially expressed genes in FTC and MTC samples	3
**Figure 2**	Heatmap of over-expressed DEGs in MTC vs Normal samples in dogs	4
**Figure 3**	Representative photomicrographs of HER2 IHC scoring	5
**Figure 4**	*ERBB2* transcript expression association with HER2 IHC scores	6
**Figure 5**	Normalized expression PIK3 pathway genes	7
**Figure 6**	Association of mutational burden and progression-free interval	8
**Figure 7**	Mutational signature analysis of canine thyroid carcinomas	9
**Figure 8**	Boxplot illustrating the distribution of MEN1 gene expression levels	10
**Figure 9**	Distribution of *TG* (A) and *CALCB* (B) gene expression levels	11
**Figure 10**	Distribution of selected fusion gene expression across all 30 samples	12
**Figure 11**	Kaplan-Meier plots for select clinical parameters	13
**Table 1**	Primer pairs used for selected variant and gene fusion validation	14
**Note 1**	Metadata on canine thyroid carcinoma whole exome sequencing	16
	Comparison of thyroid carcinoma variants identified using CanFam3.1 and CanFam4 genomes	18
	Metadata on RNAseq of canine thyroid carcinoma	20
	Comparison of differentially expressed genes between FTC and MTC using CanFam3.1 and CanFam4 genomes	21

Supplementary Figure 1: Normalized expression data of selected differentially expressed genes in FTC and MTC samples

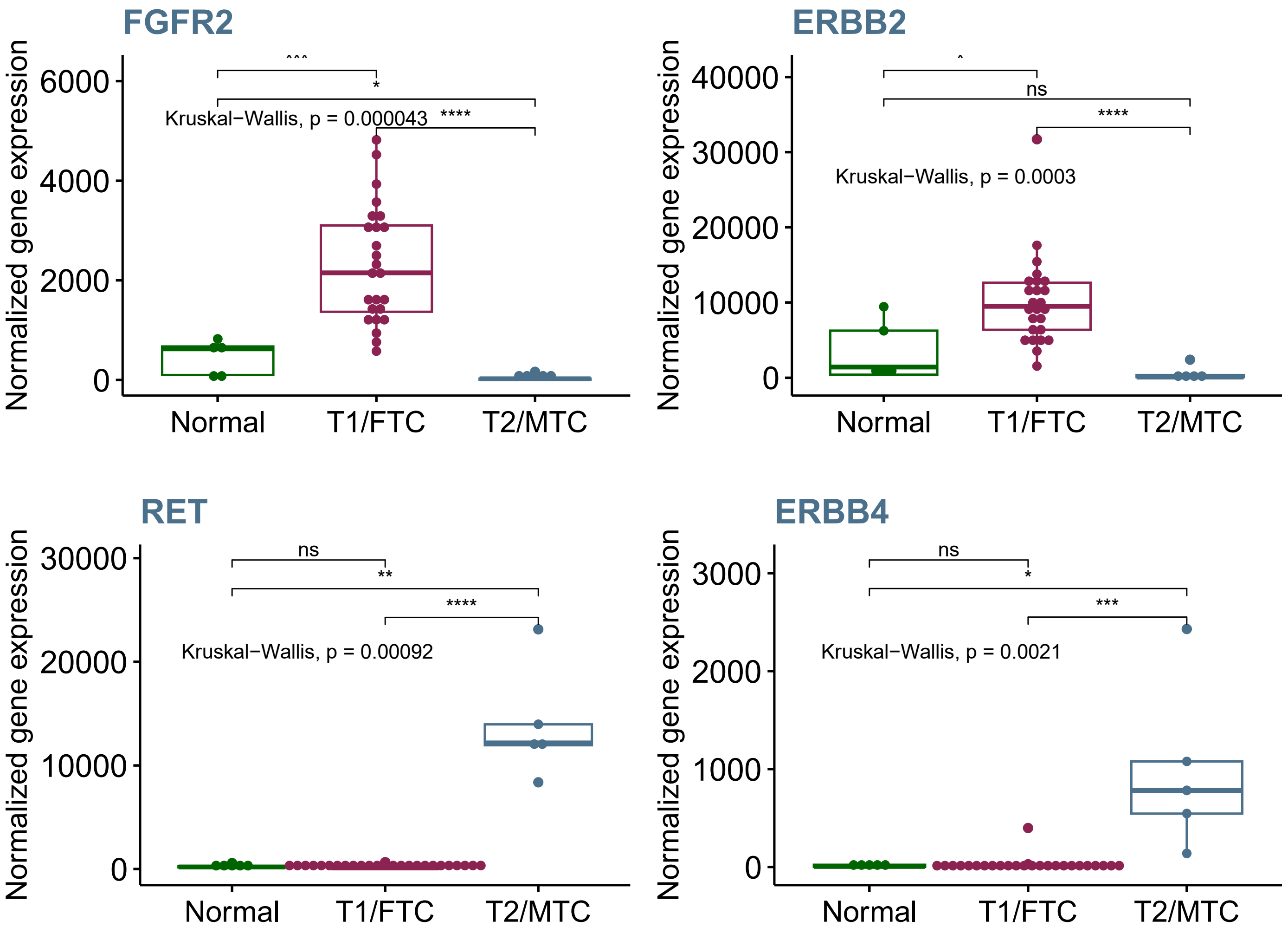

Supplementary Figure 1. Normalized expression data of selected differentially expressed genes in FTC and MTC samples. Data from five BarkBase normal samples were included.

Supplementary Figure 2: Heatmap of over-expressed DEGs in MTC vs Normal samples in dogs

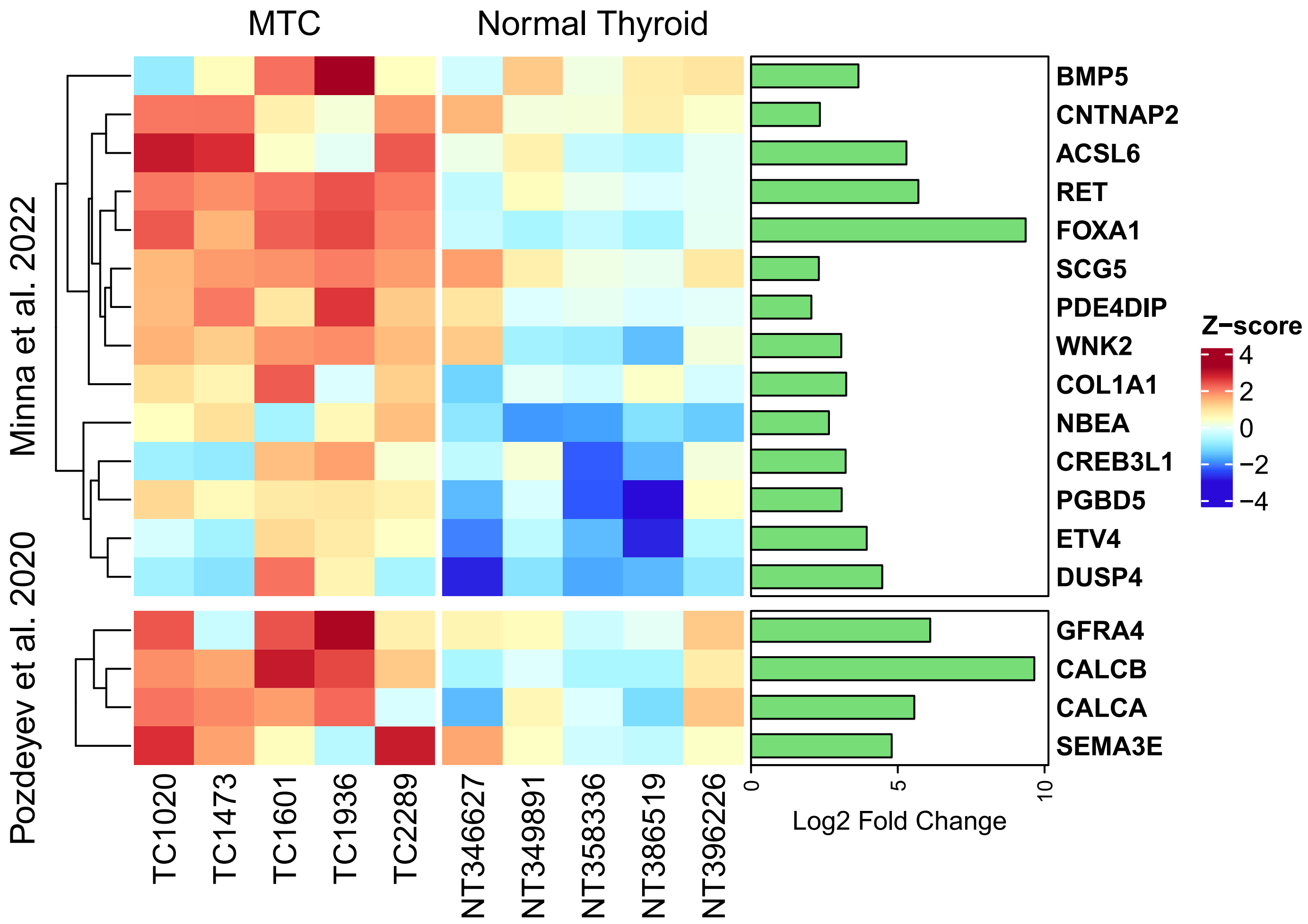

Supplementary Figure 2. Heatmap of over-expressed DEGs in MTC vs Normal samples in dogs. The genes plotted here overlap with DEGs identified in human MTC tumors relative to normal thyroid, derived from two published articles. Only cancer-related overlapping genes were plotted in the section labelled Minna et al. 2022[33].

Supplementary Figure 3: Representative photomicrographs of HER2 IHC scoring

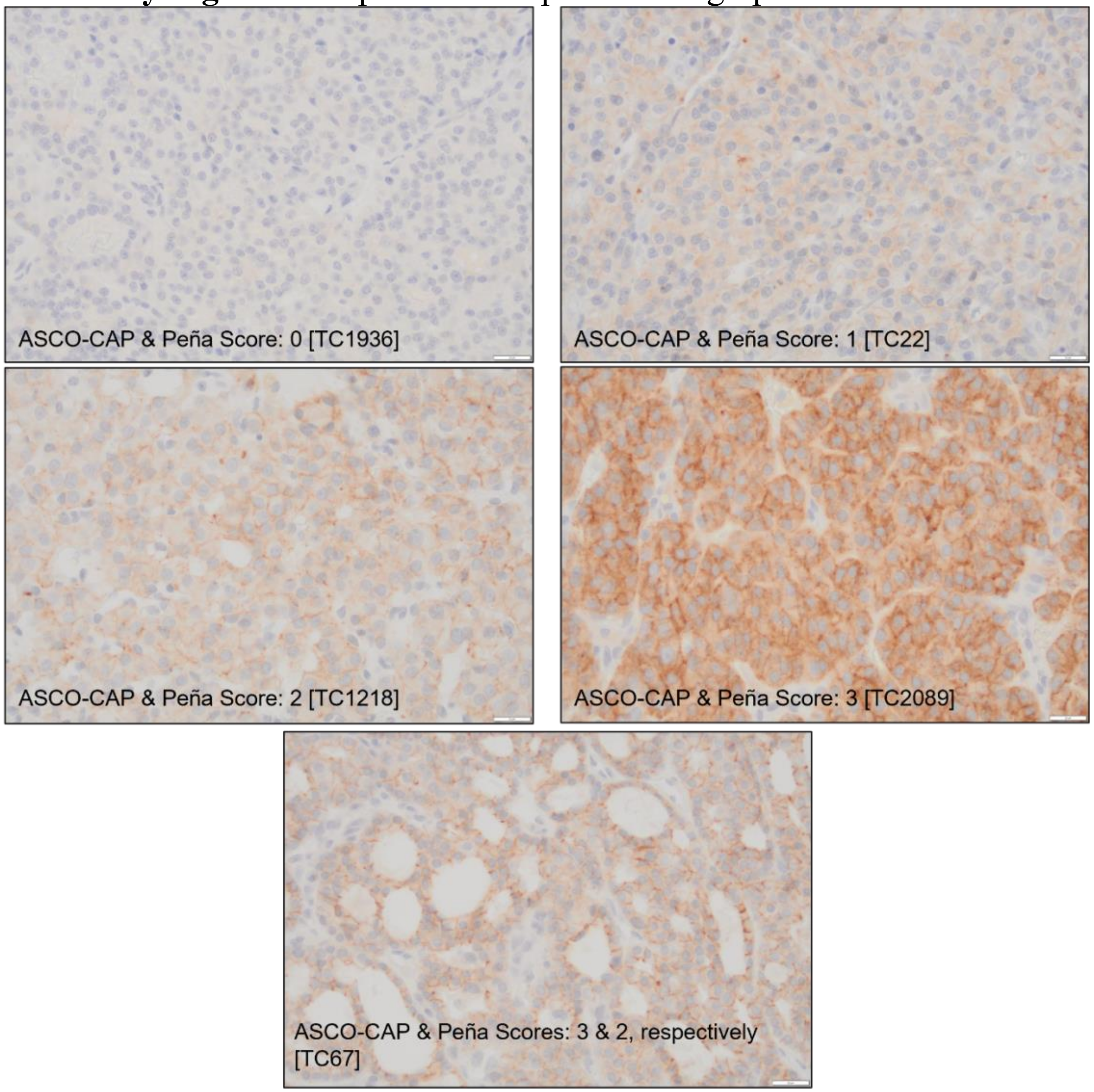

Supplementary Figure 3. Representative photomicrographs of HER2 IHC scoring, based on ASCO-CAP[34,35] and Peña[36] recommendations. 50x objective. Size bar = 20 μm.

Supplementary Figure 4: *ERBB2* transcript expression association with HER2 IHC scores

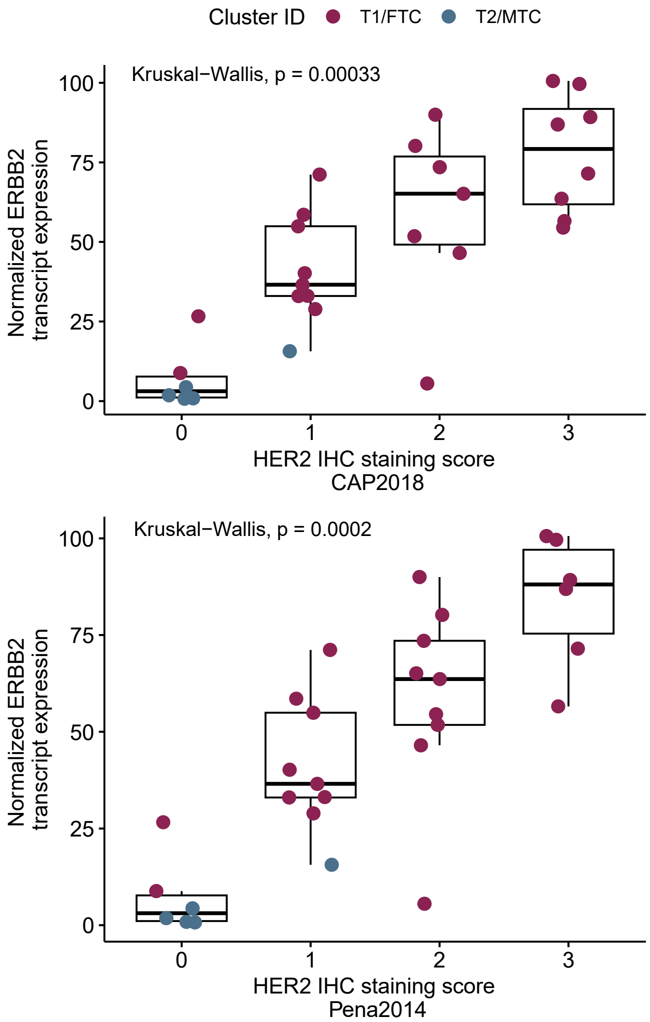

Supplementary Figure 4. Boxplot showing distribution of ERBB2 transcript expression levels across four HER2 IHC scores in canine thyroid tumors. Two different types of HER2 score were used in this analysis.

Supplementary Figure 5: Normalized expression PIK3 pathway genes

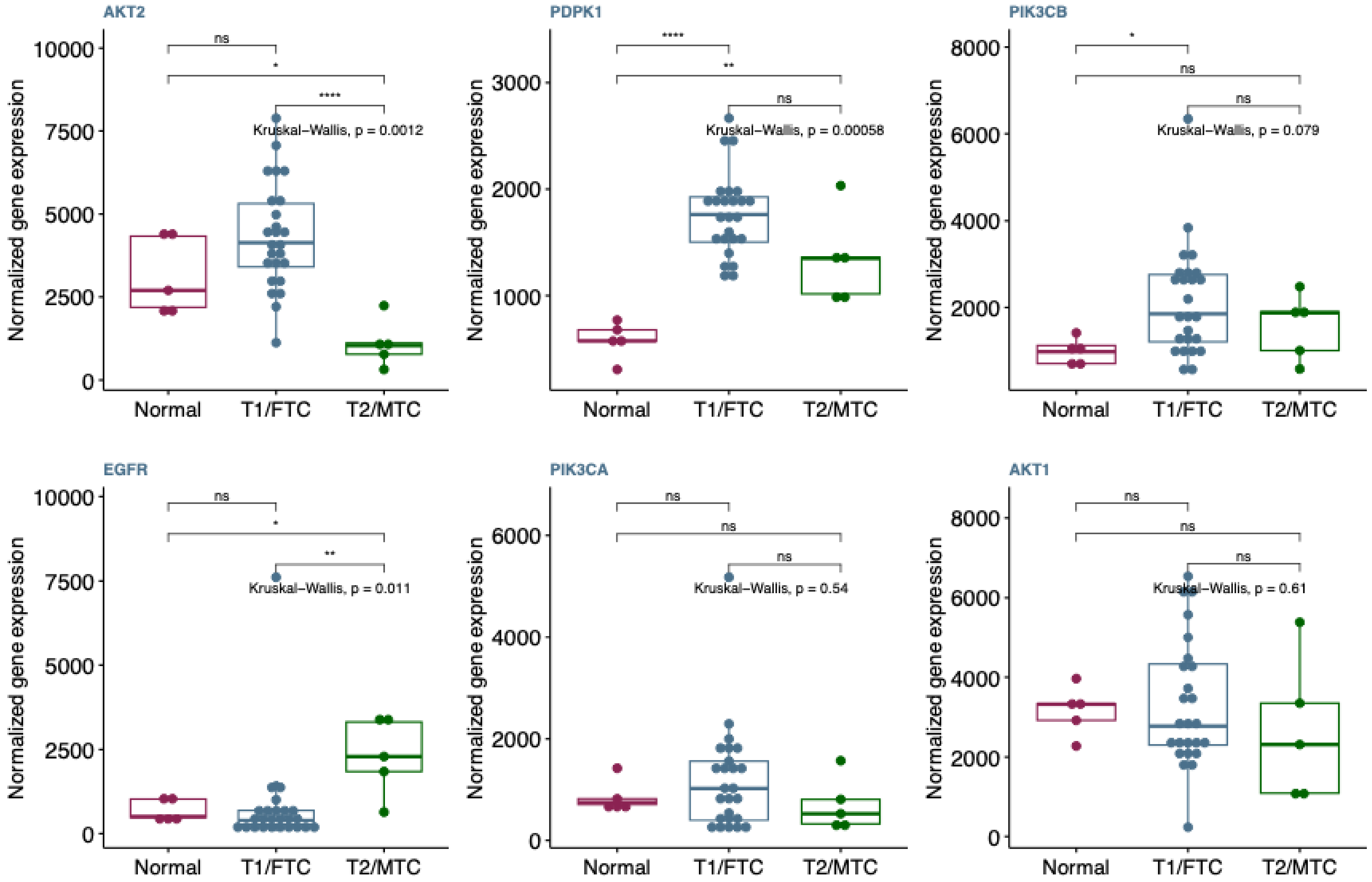

Supplementary Figure 5. Normalized expression PIK3 pathway genes. These genes have been previously reported as differentially expressed in canine thyroid tumors.

Supplementary Figure 6: Association of mutational burden and progression-free interval

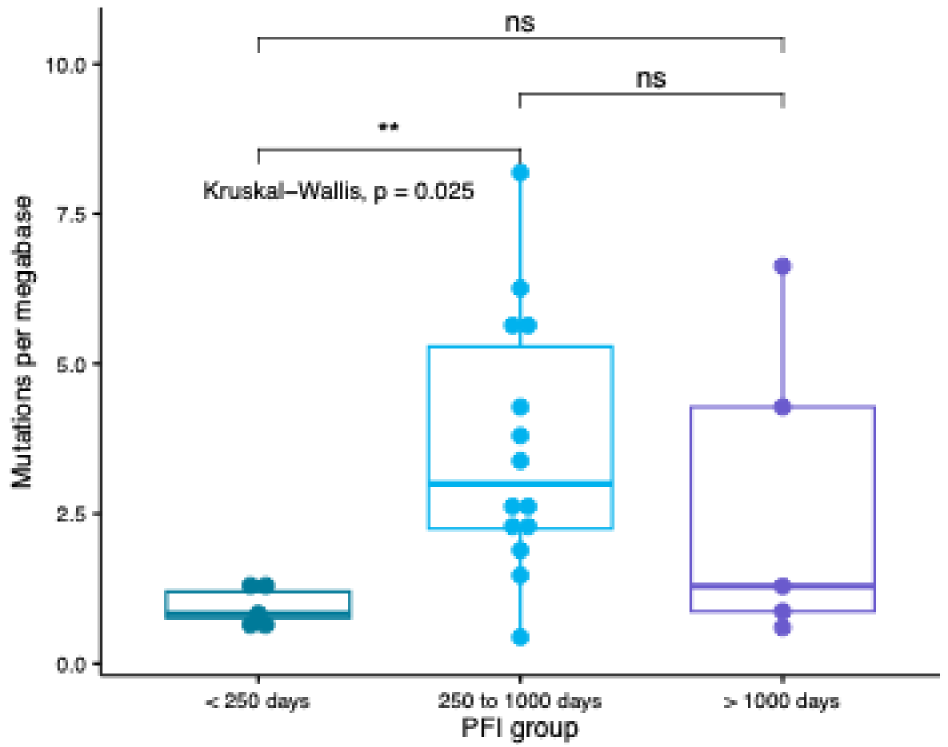

Supplementary Figure 6. Association of mutational burden (number of mutations per megabase) and progression-free interval (PFI). Dogs were segregated based on ranges of PFI. Mean number of mutations were highest in dogs with PFI range of 250 to 1000 days.

Supplementary Figure 7: Mutational signature analysis of canine thyroid carcinomas

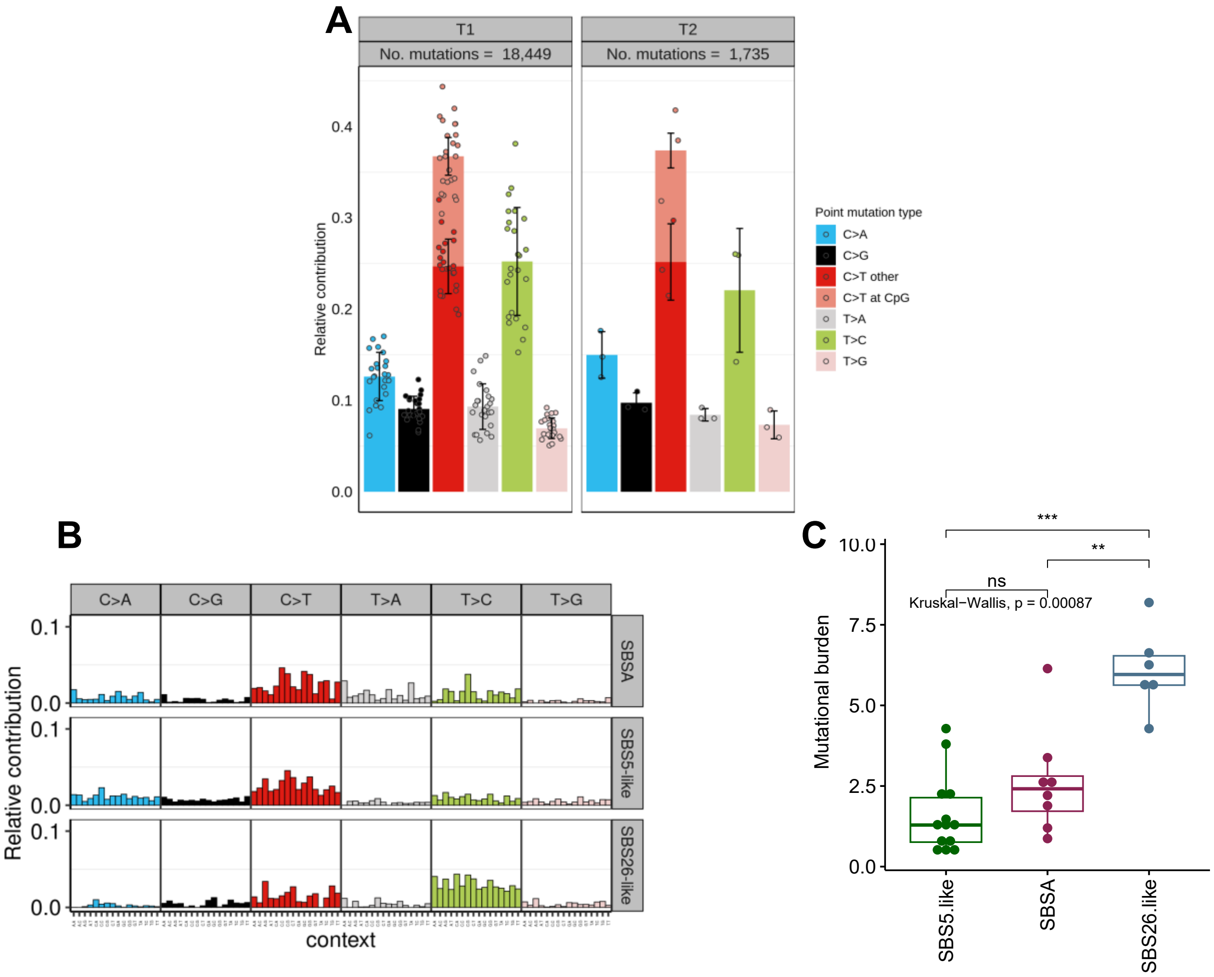

Supplementary Figure 7. Mutational signature analysis of canine thyroid carcinomas. A. Relative contribution of 6 single nucleotide substitutions in 25 FTC samples (T1 group) and 5 MTC samples (T2 group). B. Contribution of 96 trinucleotide substitutions to the three mutational signatures identified by NMF analysis of thyroid carcinoma somatic mutation profiles. C. Association of mutational burden to three identified mutational signatures. The dogs with SBS26-like mutational signature had significantly higher mutational burden compared to the other two signatures.

Supplementary Figure 8: Boxplot illustrating the distribution of MEN1 gene expression levels

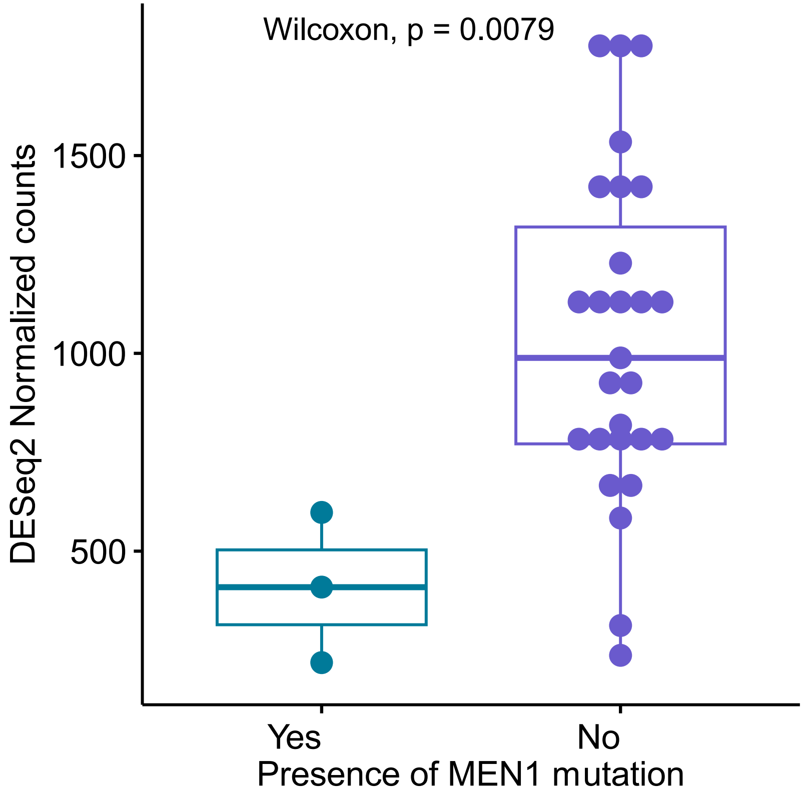

Supplementary Figure 8. Boxplot illustrating the distribution of MEN1 gene expression levels. The dogs were grouped by their mutation status (mutant vs wild-type).

Supplementary Figure 9: Distribution of TG (A) and CALCB (B) gene expression levels

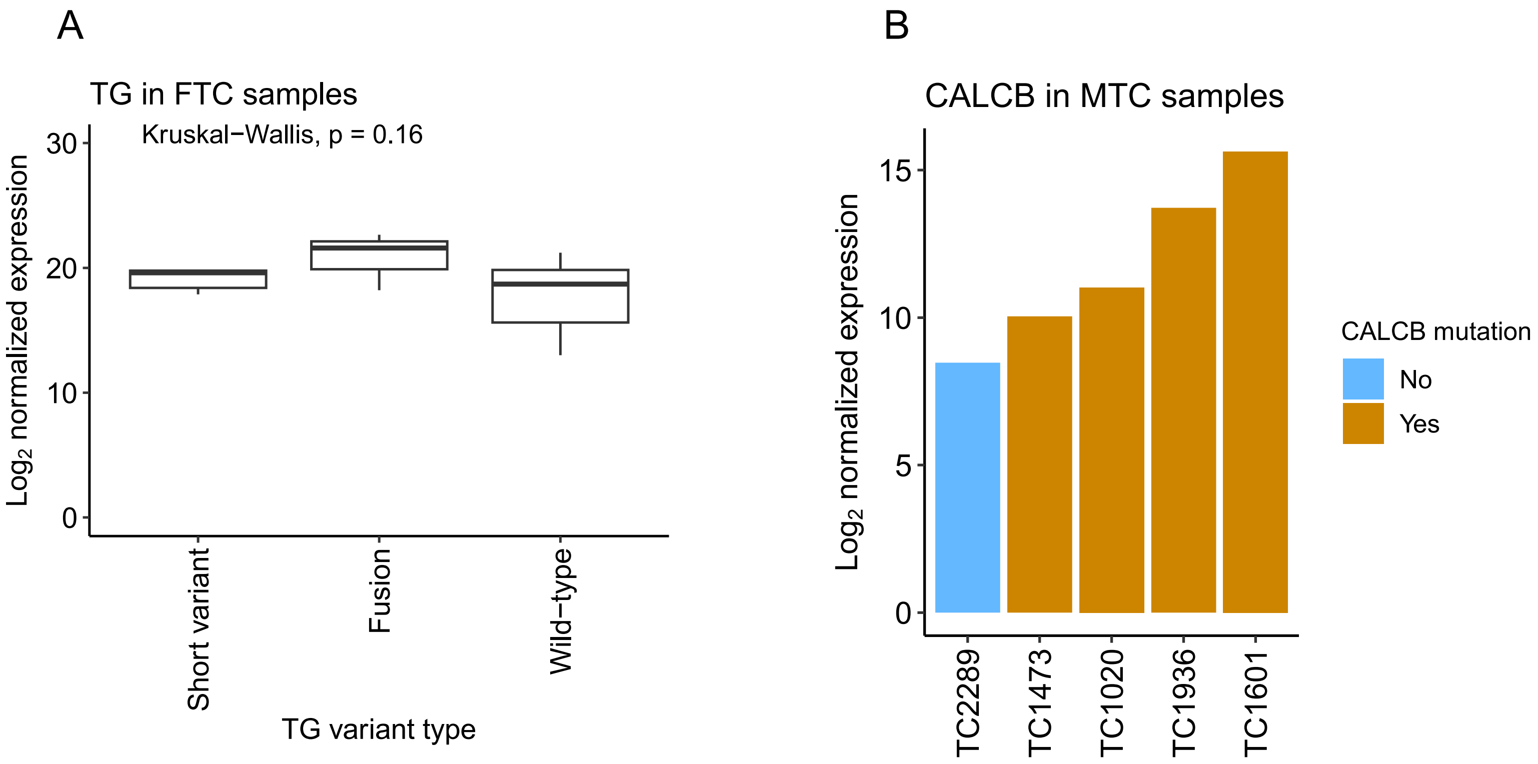

Supplementary Figure 9. Distribution of TG (A) and CALCB (B) gene expression levels. Dogs were grouped by mutation status (mutant vs wild-type) from 25 FTC (A) and 5 MTC (B) samples.

Supplementary Figure 10: Distribution of selected fusion gene expression across all 30 samples

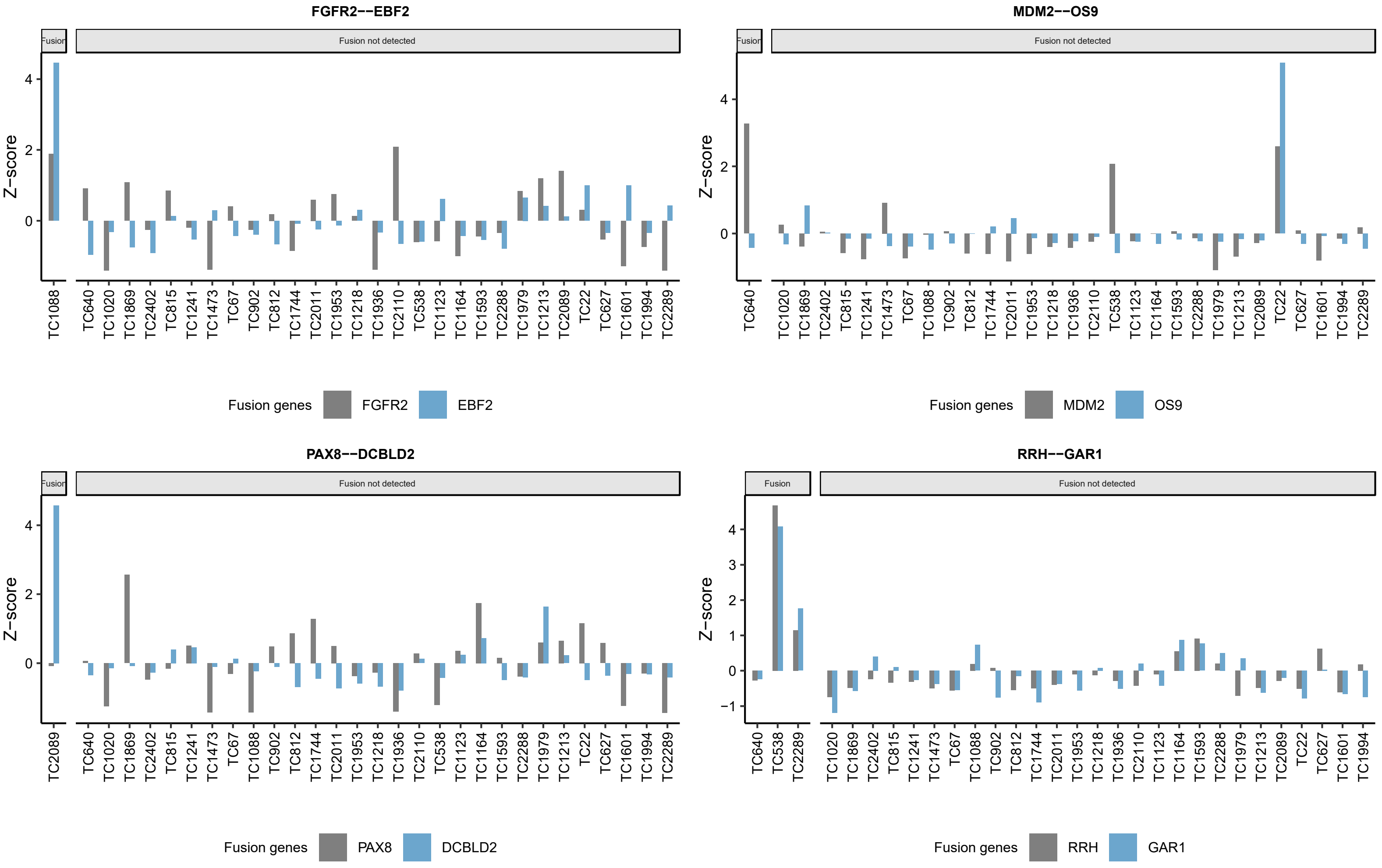

Supplementary Figure 10. Distribution of selected fusion gene expression across all 30 samples. These samples were partitioned by the presence or absence of respective fusions.

Supplementary Figure 11: Kaplan-Meier plots for select clinical parameters

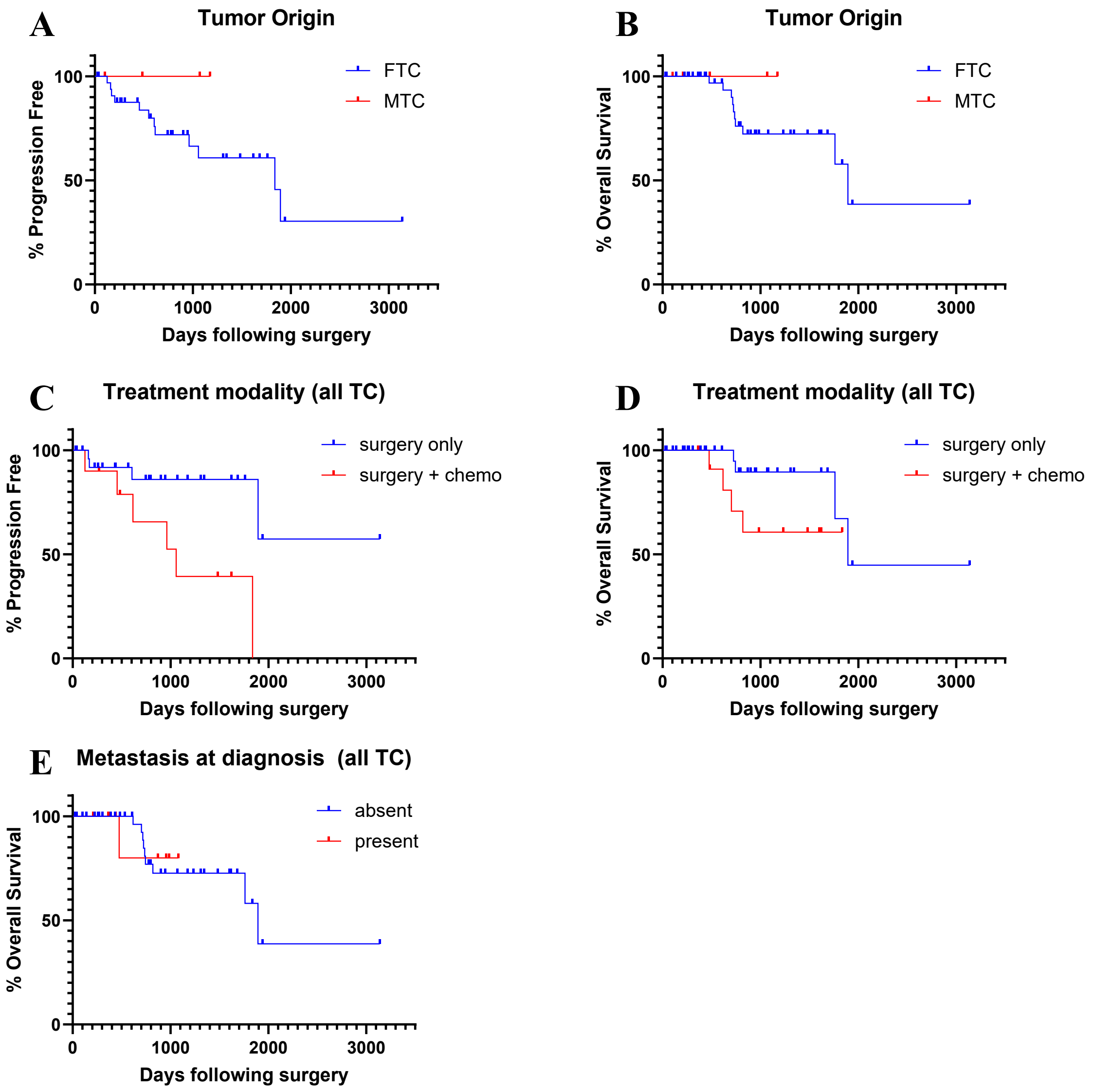

Supplementary Figure 11. Kaplan-Meier plots for select clinical parameters. These were not significant on log-rank test ($p > 0.05$).

Supplementary Table 1: Primer pairs used for select variant and gene fusion validation

Tumor	Gene or Fusion	Primer Name	Primer Sequence 5'- 3'	Confirmed
**T1088**	FGFR2 EBF2 fusion	FGFR2 FW1	CACAGAGGCCGACGTTCAAG	Yes
		EBF2 RE1	GATGCAGGGGGTAGCTTCTG	
**T2089**	PAX8--DCBLD2 fusion	PAX8 FW1	CCCTGACCCCTTCCAATACAC	Yes
		DCBLD2 RE1	GCGCACTCTCTCTCCCATC	
**T2089**	PAX8--DCBLD2 fusion	PAX8 FW2	CCCTGGACGACGGCAAGG	Yes
		DCBLD2 RE2	GCCAAGAATCCTCGTCCAGAA	
**T1164**	MEN1	MEN1 Exon7 FW	TGTGCCTTATGGGTGTGACC	Yes
		MEN1 Exon7 RE	GATAGGGTGGGGGAGAGAGG	
**T1593**	MEN1	MEN1 Exon6 FW	GCTGCTCTATGACCTGGGAC	Yes
		MEN1 Exon6 RE	TCCTGAGATGTCCCCACCTT	
**T815**	MEN1	MEN1 Exon2 FW	CTGTGGCGGACCTATCCATC	Yes
		MEN1 Exon2 RE	GGGCTCCAACCTGTGATGAA	
**TC2288**	STAT2	STAT2 Exon20 FW	TCTCTGCAACCCTTCACCAA	Yes
		STAT2 Exon20 RE	AGTAGCACCCAAAAGCCTCA	
**TC1593**	STAT2	STAT2 Exon20 FW	TCTCTGCAACCCTTCACCAA	Yes
		STAT2 Exon20 RE	AGTAGCACCCAAAAGCCTCA	
**TC627**	STAT2	STAT2 Exon20 FW	TCTCTGCAACCCTTCACCAA	Yes
		STAT2 Exon20 RE	AGTAGCACCCAAAAGCCTCA	
**T1088**	STAT2	STAT2 Exon16 FW	CAGTTCTTCTCCAGCCCTCC	Yes
		STAT2 Exon16 RE	AGCTTGTTCCTCAGCATGCT	
**T1218**	STAT2	STAT2 Exon16 FW	CAGTTCTTCTCCAGCCCTCC	Yes
		STAT2 Exon16 RE	AGCTTGTTCCTCAGCATGCT	
**T2089**	STAT2	STAT2 Exon16 FW	CAGTTCTTCTCCAGCCCTCC	Yes
		STAT2 Exon16 RE	AGCTTGTTCCTCAGCATGCT	
**T1164**	STAT2	STAT2 Exon19 F	ACAGCAGGGAGGCATGAGG	Yes
		STAT2 Exon19 R	ATCCTGATGTTCCACCCAGG	
**T2289**	STAT2	STAT2 Exon15 F	CAAGTTTCCATTGCCTGGGC	Yes
		STAT2 Exon15 R	CAAGAGTGGGAAGAGGGCAG	
**T1088**	MUC4	MUC4 Exon1 FW175	CACCAACAGTATCACAGGTCCT	Yes
		MUC4 Exon1 RE374	ACCCTGAGATGACTCTGTTGT	
**T1936**	MUC4	MUC4 Exon1 FW175	CACCAACAGTATCACAGGTCCT	No
		MUC4 Exon1 RE374	ACCCTGAGATGACTCTGTTGT	
**T538**	MUC4	MUC4 Exon1 FW175	CACCAACAGTATCACAGGTCCT	No
		MUC4 Exon1 RE374	ACCCTGAGATGACTCTGTTGT	
**T2089**	MUC4	MUC4 Exon1FW1012	CCACAGTGTCAGAAATGGCC	Yes
		MUC4 Exon1 RE1556	TGTCGTTGCTGATGTGTTGC	
**T2011**	MUC4	MUC4 Exon1FW1012	CCACAGTGTCAGAAATGGCC	Yes
		MUC4 Exon1 RE1556	TGTCGTTGCTGATGTGTTGC	
**T2288**	MUC4	MUC4 FW1850	AGATGACTCGACCCAGGACA	Yes
		MUC4 RE2371	CTGGAAGGGGACAAAGTGCT	
**T627**	MUC4	MUC4 FW1850	AGATGACTCGACCCAGGACA	No
		MUC4 RE2371	CTGGAAGGGGACAAAGTGCT	
**T1241**	KRAS	KRAS Exon3 FW	ACTGTGTTTCTCCCTTCTCAGG	Yes
		KRAS Exon3 RE	TGCATGGTAATCAGCAAAGACT	
**T1601**	KRAS	KRAS cDNA FW	TGATGGAGAAACCTGTCTCTTG	Yes
		KRAS cDNA RE	GGACCATAGGTACATCTTCAGAGT	
**All 60 samples and CTAC cell line**	ERBB2^{V659E}	ERBB2 FW	CTTCGCTTGATCAGGATGC	Yes for only CTAC
		ERBB2 RE	AAACATCAGGGACCTGCCTA	

Supplementary Note 1: Metadata on canine thyroid carcinoma Whole Exome Sequencing

1. Sequencing and mapping statistics

Whole exome sequencing (WES) was conducted on 27 tumors and matched normal samples. The total number of reads ranged from 27.8–41.7 million in normal samples and 66.2–90.6 million in tumor samples (**Figure A1**). The average mapping rates were 99.1% (±0.29%) and 99.2% (±0.28) in samples mapped against CanFam3.1 and CanFam4, respectively (**Figure A2**). The tumor median coverage/depth of mapping against two genomes, CanFam3.1 and CanFam4, were 220X (±27.8X) and 215.6X (±27.4X), and the matched normal median coverage/depth were 100.8X (±10.9X) and 111.9 (±12.3X), respectively (**Figure A3**). The protein-coding mutational burden (total protein coding variants per 43.5 MB exome capture) ranged from 0.44 (TC640) to 8.19 (TC627) and 0.64 (TC1869) to 10.59 (TC627) when mapped against the CanFam3.1 and CanFam4 genomes, respectively.

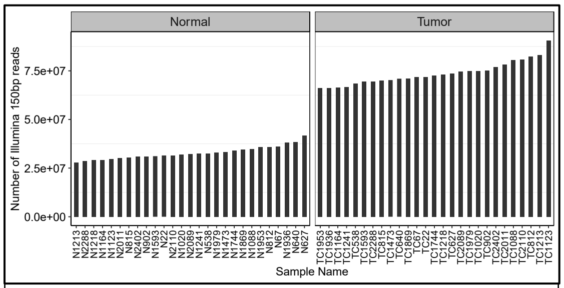

Figure A1. Total number of raw reads from Illumina sequencing of canine thyroid carcinoma tumors and matched normals.

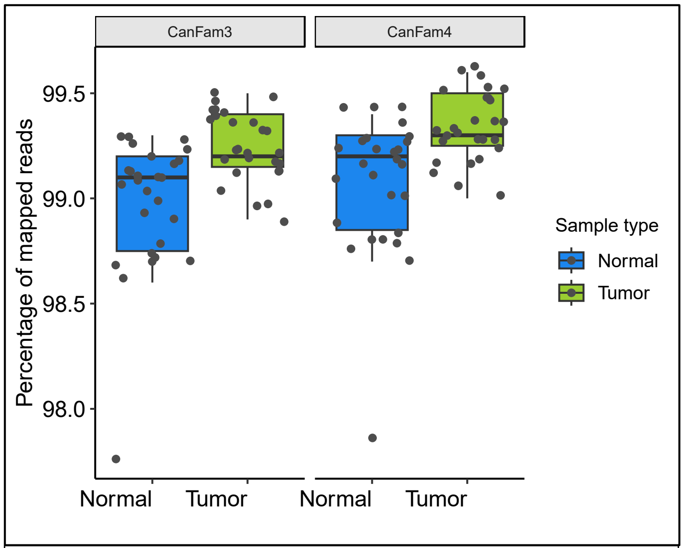

Figure A2. Percentage of mapped reads in normal and tumor samples from Whole Exome Sequencing. Similar mapping rates were observed in both cases.

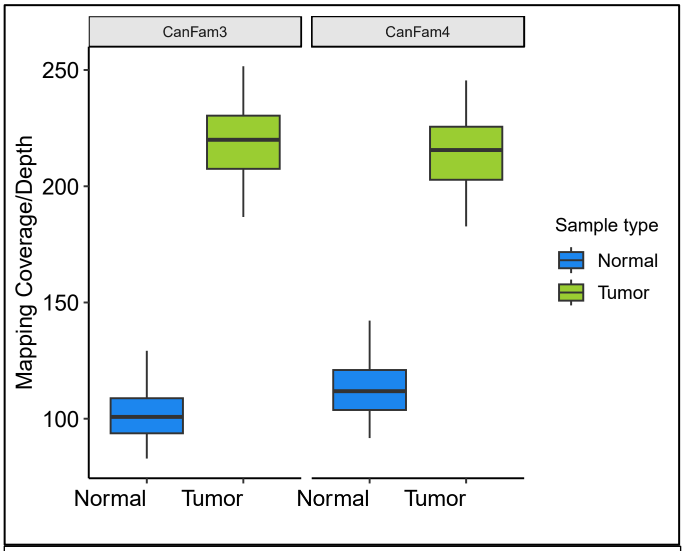

Figure A3. Whole exome sequencing coverage in normal and tumor samples. Mapping against CanFam3.1 and CanFam4 showed similar depth.

2. Variant statistics

The total number of Mutect2 PASS variants identified using CanFam3.1 and CanFam4 genome were very similar. The number of variants (SNV and INDELs) ranged from 410 (TC1020) to 2,110 (TC627) in the analysis using CanFam3.1 genome and were 278 (TC1020) and 2,125 (TC627) when using CanFam4 genome (Figure A4). On average, 11% of CanFam3.1 PASS variants were located within coding regions, while 16% of CanFam4 variants were identified in coding regions. The number of coding variants ranged from 19 (TC640) to 356 (TC627) in the analysis using CanFam3.1 genome, and from 28 (TC1020) and 460 (TC627) in the analysis using CanFam4 genome. Approximately, 5% of these protein coding variants were identified in cancer genes irrespective of the genome used for variant calling.

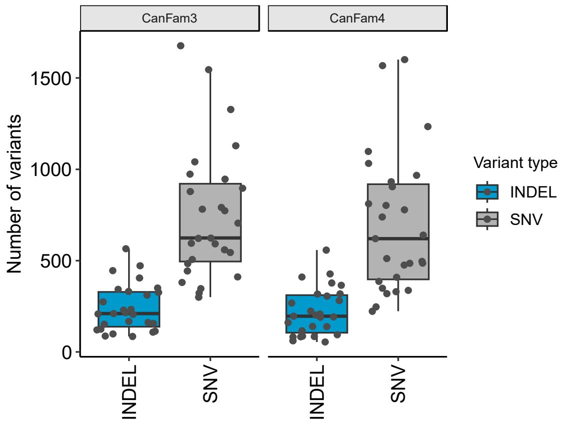

Figure A4. Whole exome sequencing coverage in normal and tumor samples. Mapping against CanFam3.1 and CanFam4 showed similar depth.

3. Coding variant mutation types

The distribution of mutation types was plotted in **Figure A5**. The most common type of mutation identified was missense mutation followed by mutations in UTR regions. On average 68% and 61% of all coding mutations were identified as missense using CanFam3.1 and CanFam4 as reference genomes, respectively. The difference was observed in the UTR regions, where significantly more variants were identified when CanFam4 was used compared to CanFam3.1 (**Figure A6**).

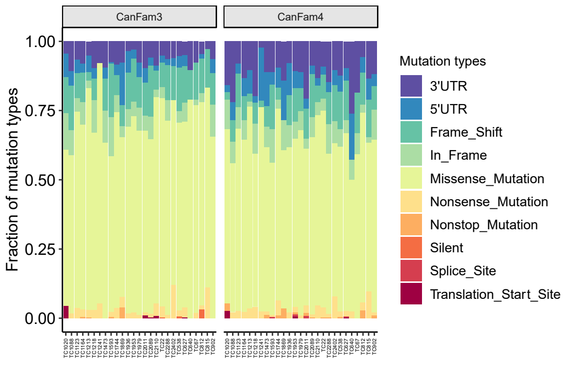

Figure A5. Distribution of coding mutation types in samples using CanFam3.1 and CanFam4 as reference genomes. Missense mutations were the most common type of short variant as identified in both pipelines.

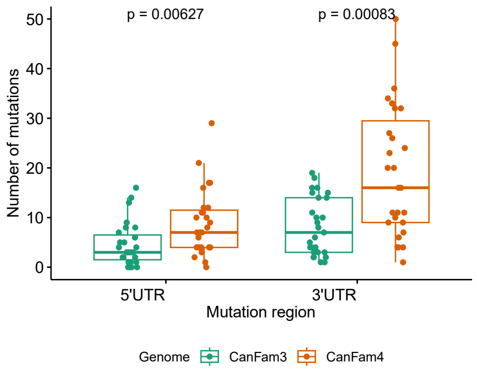

Figure A6. Boxplots representing the number of mutations at UTR regions from analyses using CanFam3.1 and CanFam4 as reference genomes.

Supplementary Note 1: Comparison of thyroid carcinoma variants identified using CanFam3.1 and CanFam4 genomes

Using the same pipeline with different reference genomes, more PASS variants were identified from CanFam3.1 (n = 26,695) compared to CanFam4 (n = 24,722) genome from 27 thyroid tumor samples. However, relatively more CDS variants were identified using CanFam4 (n=6,169) compared to CanFam3.1 (n=4,909) genome. Among all these coding sequences, 5.5% and 6.8% of variants were synonymous/silent mutations as derived using CanFam3.1 and CanFam4 genomes, respectively.

Looking into the non-synonymous cancer and thyroid gland-specific gene variants, we have identified 100 genes with variants from CanFam3.1 genome and 107 genes from CanFam4 genome. The gene variants that were identified in either CanFam3.1 or CanFam4 are listed in **Table A1**.

Table A1. Cancer gene variants detected using CanFam3 or CanFam4 genomes.

Tumor Sample Barcode	Gene name	Cancer Gene Type	Depth	Alternate Allele Count	Tumor allelic frequency	HGVSp	Genome
TC1218	ARAF	Oncogene	82	8	0.081	p.T235P	CanFam3
TC627	DEK	Oncogene	35	2	0.081	p.P280A	CanFam3
TC1744	ERBB2	Oncogene	232	4	0.021	p.H42R	CanFam3
TC1241	KDM5A	Oncogene	24	2	0.115	p.I241T	CanFam3
TC1213	DDX3X	TSG	40	2	0.071	p.S476T	CanFam3
TC1953	KDM6A	TSG	27	5	0.206	p.G1243S	CanFam3
TC1164	NF2	TSG	24	2	0.114	p.*598Yext*?	CanFam3
TC1123	RECQL	TSG	68	3	0.056	p.N285K	CanFam3
TC22	SH2B3	TSG	35	2	0.081	p.R242L	CanFam3
TC538	BRD3	Unassigned	155	4	0.031	p.M125T	CanFam3
TC1744	CARM1	Unassigned	35	4	0.135	p.N700Pfs*?	CanFam3
TC1164	CDH10	Unassigned	371	4	0.013	p.H773R	CanFam3
TC2089	KAT6B	Unassigned	47	3	0.081	p.E1102V	CanFam3
TC902	MN1	Unassigned	281	7	0.027	p.Q315dup	CanFam3
TC627	NONO	Unassigned	15	2	0.176	p.R292H	CanFam3
TC627	SDC4	Unassigned	14	3	0.25	p.A87G	CanFam3
TC2110	MN1	Unassigned	107	5	0.052	p.Q315del	CanFam3
TC2011	SPECC1	Unassigned	135	4	0.035	p.S9I	CanFam3
TC2011	TPR	Unassigned	31	2	0.091	p.L260Q	CanFam3
TC22	CD276	Oncogene	105	5	0.054	p.H449R	CanFam4
TC627	EIF4E	Oncogene	26	2	0.107	p.N168D	CanFam4
TC538	GAB2	Oncogene	19	2	0.144	p.K20E	CanFam4
TC1936	H1-4	Oncogene	340	5	0.018	p.A10P	CanFam4
TC1213	H3-3A	Oncogene	23	2	0.12	p.P67S	CanFam4
TC1213	H3-3A	Oncogene	23	2	0.119	p.K65T	CanFam4
TC2288	MAPK3	Oncogene	65	6	0.102	p.L252P	CanFam4
TC538	STK19	Oncogene	28	6	0.222	p.R205Q	CanFam4
TC1593	STK19	Oncogene	62	5	0.093	p.R205Q	CanFam4
TC2089	STK19	Oncogene	75	10	0.141	p.R205Q	CanFam4
TC1473	STK19	Oncogene	41	5	0.137	p.R205Q	CanFam4
TC627	STK19	Oncogene	107	4	0.046	p.T254M	CanFam4
TC1164	STK19	Oncogene	68	10	0.155	p.R205Q	CanFam4
TC2288	STK19	Oncogene	60	8	0.143	p.R205Q	CanFam4
TC1473	STK19	Oncogene	71	6	0.095	p.I233T	CanFam4
TC1979	STK19	Oncogene	102	3	0.038	p.R250H	CanFam4
TC1123	XBP1	Oncogene	156	5	0.037	p.L189P	CanFam4
TC1123	XBP1	Oncogene	148	5	0.039	p.S193P	CanFam4
TC1241	XBP1	Oncogene	132	6	0.05	p.L18M	CanFam4
TC1088	ARID1A	TSG	715	18	0.024	p.N1951S	CanFam4
TC1213	ERCC5	TSG	19	2	0.143	p.L701K	CanFam4
TC2288	H1-3	TSG	172	4	0.029	p.T168A	CanFam4
TC1213	KMT2D	TSG	206	4	0.024	p.Q3718*	CanFam4
TC22	NPM1	TSG	39	3	0.097	p.R101Q	CanFam4
TC1744	PDS5B	TSG	65	5	0.088	p.Q316R	CanFam4
TC2011	RBM10	TSG	106	4	0.045	p.D229E	CanFam4
TC2288	SOX17	TSG	77	4	0.063	p.E264_V267del	CanFam4
TC1123	DKK1	Unassigned	89	3	0.044	p.E94_E95insL	CanFam4
TC1123	DKK1	Unassigned	88	3	0.044	p.S92_P93delinsRT	CanFam4
TC2110	ERC1	Unassigned	72	4	0.066	p.Q226K	CanFam4
TC1218	H1-2	Unassigned	692	7	0.011	p.V57L	CanFam4
TC2288	H2AC11	Unassigned	82	4	0.058	p.S129G	CanFam4

Tumor Sample Barcode	Gene name	Cancer Gene Type	Depth	Alternate Allele Count	Tumor allelic frequency	HGVSp	Genome
**TC2089**	H3-3B	Unassigned	488	6	0.014	p.L49Rfs*12	CanFam4
**TC2288**	H3-3B	Unassigned	317	8	0.028	p.T81Lfs*49	CanFam4
**TC2089**	H3-3B	Unassigned	437	7	0.017	p.R54C	CanFam4
**TC2011**	MUC4	Unassigned	124	5	0.045	p.E352Q	CanFam4
**TC1241**	MUC4	Unassigned	11	2	0.231	p.L406P	CanFam4
**TC812**	MUC4	Unassigned	26	3	0.143	p.L406P	CanFam4
**TC1953**	MUC4	Unassigned	196	3	0.02	p.T723I	CanFam4
**TC1473**	MUC4	Unassigned	16	2	0.167	p.S147T	CanFam4
**TC627**	MUC4	Unassigned	67	7	0.111	p.E105A	CanFam4
**TC2089**	MUC4	Unassigned	54	6	0.124	p.L406P	CanFam4
**TC2089**	MUC4	Unassigned	166	4	0.029	p.P510L	CanFam4
**TC2110**	MUC4	Unassigned	28	4	0.166	p.L406P	CanFam4
**TC1953**	MUC4	Unassigned	200	3	0.02	p.M724I	CanFam4
**TC1164**	MUC4	Unassigned	610	18	0.026	p.G85S	CanFam4
**TC538**	MUC4	Unassigned	98	8	0.084	p.S492R	CanFam4
**TC1088**	MUC4	Unassigned	325	11	0.034	p.G85S	CanFam4
**TC1936**	MUC4	Unassigned	208	8	0.039	p.N498S	CanFam4
**TC1936**	MUC4	Unassigned	66	7	0.117	p.Y110H	CanFam4
**TC1936**	MUC4	Unassigned	63	6	0.108	p.M111S	CanFam4
**TC1593**	MUC4	Unassigned	49	3	0.078	p.H811Q	CanFam4
**TC1473**	MUC4	Unassigned	16	2	0.167	p.D146G	CanFam4
**TC1936**	MUC4	Unassigned	392	6	0.017	p.P773L	CanFam4
**TC2288**	MUC4	Unassigned	550	9	0.017	p.R644S	CanFam4
**TC902**	MUC4	Unassigned	57	4	0.084	p.I422T	CanFam4
**TC627**	MUC4	Unassigned	532	7	0.015	p.R644S	CanFam4
**TC1473**	POLG	Unassigned	195	7	0.034	p.P49Q	CanFam4
**TC1213**	POLG	Unassigned	228	9	0.041	p.P46Q	CanFam4
**TC1744**	POLG	Unassigned	175	6	0.039	p.P50Q	CanFam4
**TC627**	RAP1GDS1	Unassigned	14	2	0.188	p.V230E	CanFam4
**TC1088**	SIRPA	Unassigned	13	8	0.6	p.H81G	CanFam4

Supplementary Note 1: Metadata on RNAseq of canine thyroid cancer

RNAseq was conducted on 30 canine thyroid tumor samples. The total number of Illumina sequencing reads ranged from 103.9–232.7 million in these tumor samples (**Figure A7**). The average mapping rates were 91.0% (±2.3%) and 93.2% (±2.1%) when mapped against CanFam3.1 and CanFam4, respectively. Although the total number of reads mapped to CanFam4 was higher than to CanFam3.1, the median coverage depth of the coding regions (or exons) was significantly greater in CanFam3.1 than in CanFam4. (**Figure A8**).

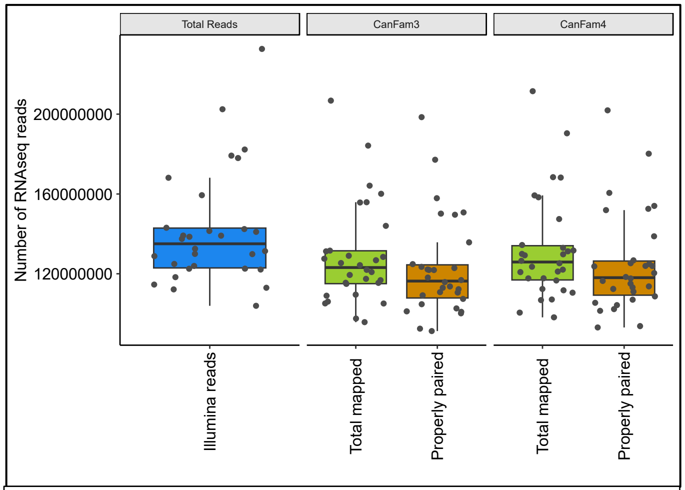

Figure A7. Boxplots representing the distribution of total reads obtained following Illumina sequencing. The total reads mapped and properly paired against CanFam3.1 and CanFam4 genomes were also plotted here.

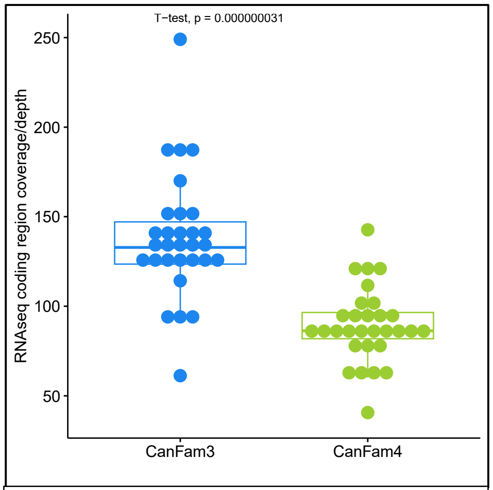

Figure A8. Boxplots representing the distribution of RNAseq coding region coverage when mapped against CanFam3.1 and CanFam4.

Supplementary Note 1: Comparison of differentially expressed genes between FTC and MTC using CanFam3.1 and Canfam4 genomes

We performed differential analysis between FTC and MTC using gene expression data from both CanFam3.1 and CanFam4 genomes. A total of 2,389 and 3,334 differentially expressed genes (DEGs) were identified using CanFam3.1 and CanFam4 genomes, respectively. However, the CanFam4 pipeline failed to identify genes with symbols or names for over 41% of DEGs, a significant limitation for downstream pathway analysis, while the CanFam3.1 pipeline only missed gene symbols for 28% of DEGs. Forty-seven percent of DEGs overlapped between the two pipelines, but this overlap increased to 77% when only genes with known symbols were considered (**Figure A9**). The list of DEGs that were identified by using either CanFam3.1 or CanFam4 are listed in **Table A2**.

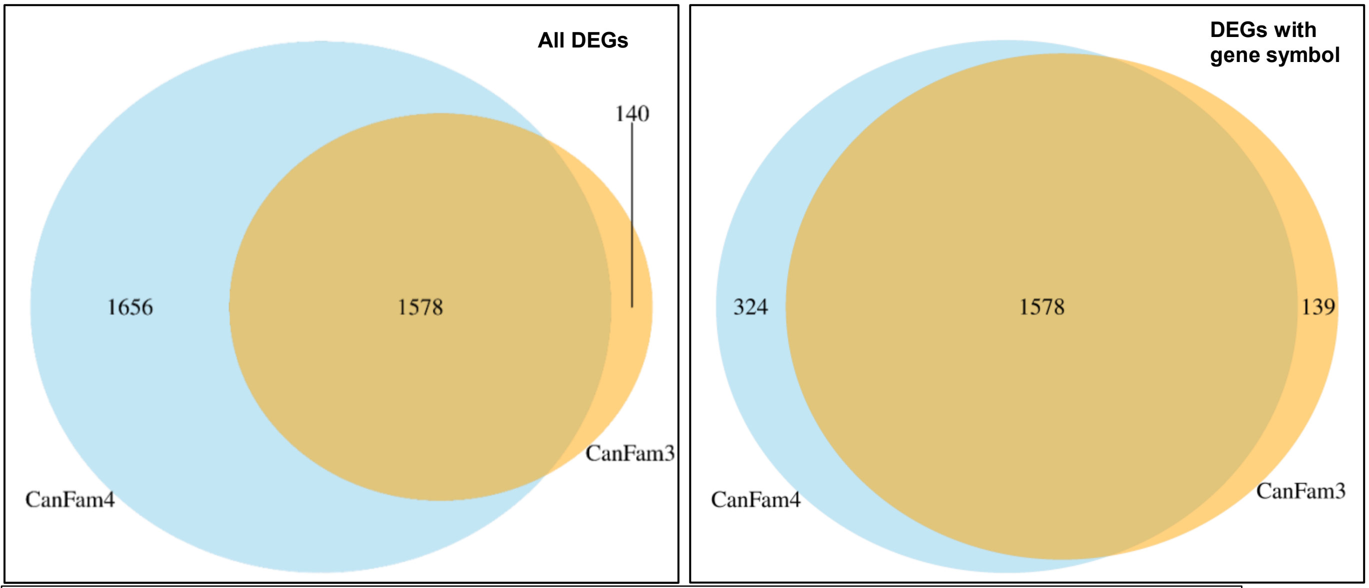

Figure A9. Venn diagrams showing the overlap of DEGs genes identified using CanFam3.1 and CanFam4 genomes.

Table A2. List of DEGs identified using either CanFam3.1 or CanFam4 genome.

Gene symbol	Log2 fold change	Adjusted p-value	Genome
ABHD12B	-2.0294239	0.00385353	CanFam3
ABTB2	-2.1750871	0.01050449	CanFam3
ACTRT1	-2.1806577	0.02812997	CanFam3
AHR	-2.0736517	2.2057E-08	CanFam3
AMPD3	-3.4116655	2.8768E-10	CanFam3
AOC1	-3.0301835	2.0639E-07	CanFam3
APBB1	-2.3878394	1.0722E-10	CanFam3
ARHGDIG	-2.7738064	0.00239007	CanFam3
ARSH	-4.2889948	1.0401E-05	CanFam3
BTN2A2	-2.0641596	0.02439923	CanFam3
C1QTNF7	-2.0396162	0.01171312	CanFam3
C20orf204	-2.842168	5.0176E-05	CanFam3
C31H21orf58	-3.6947738	1.4783E-08	CanFam3
C5orf58	-3.4546469	6.8763E-07	CanFam3
C8orf88	-4.4001797	1.8452E-10	CanFam3
C9H17orf99	-4.677329	1.3267E-14	CanFam3
CCDC154	-3.4235199	0.00011537	CanFam3
CCKAR	-7.5236018	5.7983E-08	CanFam3
CCN4	-2.02649	0.00527781	CanFam3
CD7	-2.0630132	0.00040739	CanFam3

CDK5R1	-3.118698	3.6244E-14	CanFam3
CFTR	-8.3221658	6.8109E-07	CanFam3
CLMP	-2.9777211	7.3141E-05	CanFam3
CNGB1	-3.3009352	0.00025312	CanFam3
DAO	-7.2476015	5.3189E-27	CanFam3
DGKE	-2.0351655	6.5922E-10	CanFam3
DLGAP3	-2.0098078	0.01367963	CanFam3
DNAI1	-2.3970211	0.00240485	CanFam3
FAM163B	-2.2038636	0.00324119	CanFam3
FAM71C	-2.0146622	0.00929241	CanFam3
FNDC4	-2.0051737	0.01187772	CanFam3
FSD2	-2.4590712	3.3455E-05	CanFam3
GABRR2	-5.8279115	2.9571E-14	CanFam3
GCKR	-2.8031237	2.8097E-14	CanFam3
GDF15	-2.4813712	0.01193277	CanFam3
GLRB	-2.0444008	0.03116223	CanFam3
GPR137C	-2.112064	0.00037513	CanFam3
GRAP2	-2.1763883	0.00169525	CanFam3
GRID2	-3.273476	0.04464276	CanFam3
GUCY1A2	-2.1155788	5.6377E-05	CanFam3
HLA-DQB1	-2.0072062	0.02425856	CanFam3
HTR7	-2.8710034	1.8986E-08	CanFam3
ITIH1	-3.1173385	1.9363E-06	CanFam3
KRT14	-3.6087258	0.0083395	CanFam3
LINGO4	-2.6207595	0.01256302	CanFam3
MESP2	-2.2245924	0.04070979	CanFam3
MYCL	-3.0423454	2.3111E-06	CanFam3
NECTIN3	-2.1155276	0.01752171	CanFam3
NPAS4	-2.4382376	0.00232997	CanFam3
NPPA	-2.3046305	0.00255929	CanFam3
NTSR2	-6.5379136	1.1458E-11	CanFam3
OSGIN1	-3.0809401	0.00015496	CanFam3
PAMR1	-2.3450478	0.00426179	CanFam3
PCDHA5	-6.1165167	3.8431E-12	CanFam3
PDE3B	-5.1666124	9.2847E-20	CanFam3
PDE4DIP	-2.3974142	2.28E-06	CanFam3
PLAC8	-2.6284164	1.2649E-06	CanFam3
PLAC8A	-2.7039425	0.00122767	CanFam3
PPM1E	-2.0666585	0.00019511	CanFam3
PRELID3A	-2.1217036	0.00022711	CanFam3
RADIL	-4.1290756	4.417E-24	CanFam3
RBFOX3	-3.4525824	1.0828E-06	CanFam3
RPS6KL1	-2.3001941	1.2571E-05	CanFam3
RTN4	-2.0233072	3.3416E-05	CanFam3
S100A5	-2.4303772	0.00016679	CanFam3
SAA1	-3.8403348	0.01137159	CanFam3
SCIN	-2.5606132	0.00430273	CanFam3
SEC14L4	-5.7706408	2.2057E-08	CanFam3
SERPINA3	-5.1686358	0.00044673	CanFam3
SERTAD4	-2.064451	0.0142564	CanFam3
SLC25A44	-2.3603125	9.9002E-06	CanFam3
SMTNL2	-2.2984318	0.00202747	CanFam3
SOX5	-2.3138233	0.0044849	CanFam3
SPATA46	-3.1306141	4.8555E-06	CanFam3
SUSD4	-2.4709197	0.04685741	CanFam3
TENM3	-4.5234536	8.3174E-06	CanFam3
TENT5A	-2.0622421	1.2161E-06	CanFam3
TMEM249	-2.1854182	0.00038202	CanFam3
TRIM67	-6.5221971	1.736E-20	CanFam3
WNT16	-7.3755005	1.4486E-13	CanFam3
ZBP1	-2.1678886	0.00019651	CanFam3
ZBTB7C	-2.3674431	0.00170212	CanFam3
ZMAT5	-2.279991	3.9907E-12	CanFam3
ZNF536	-3.7691613	0.00213549	CanFam3
ACTRT3	3.57871671	4.7467E-05	CanFam3
ANKRD9	2.49881772	2.2508E-05	CanFam3
AQP4	2.30130228	0.02661649	CanFam3
C12H6orf15	6.11588994	0.00014658	CanFam3
C17orf113	2.46931836	1.7934E-06	CanFam3
C18H11orf80	4.56442466	0.0016655	CanFam3
C8orf89	3.79991712	4.2607E-06	CanFam3
CD82	2.00825847	0.00018837	CanFam3
CEP126	2.00324703	0.0006195	CanFam3
CLEC20A	2.00730228	0.00465832	CanFam3
COL8A1	3.14604608	5.6477E-08	CanFam3
DEPDC1B	3.5136982	3.1074E-13	CanFam3
DNAI3	3.94567159	0.00093295	CanFam3
DPEP2	3.57910699	2.0969E-07	CanFam3
ELFN2	3.34141532	0.0022368	CanFam3
FAM110C	3.49892364	0.02376723	CanFam3
GCNT7	3.06343233	2.8442E-13	CanFam3
GPR101	3.95943369	0.03175109	CanFam3
JAM2	3.36781013	1.6632E-05	CanFam3
KMO	2.74918019	0.0003803	CanFam3
LBP	4.3837244	5.2832E-05	CanFam3
LHPP	2.05323315	0.00025328	CanFam3
LRRC7	2.31194418	0.04756057	CanFam3

LRRK1	2.02318374	0.00033766	CanFam3
MSRB3	2.09268839	9.2021E-07	CanFam3
MT3	5.81307975	3.1035E-12	CanFam3
MYBL2	2.22798508	9.3456E-06	CanFam3
MYBPC1	3.2782271	0.00202424	CanFam3
MYL2	2.7440499	0.00240485	CanFam3
MYL3	2.63212643	0.00037128	CanFam3
NKX6-2	5.67182404	0.0011218	CanFam3
NR0B1	5.52807747	0.00057576	CanFam3
PHKG1	2.08961821	5.9095E-05	CanFam3
PNMA6A	5.15996602	3.6943E-05	CanFam3
PRSS12	2.29990585	2.6654E-06	CanFam3
RAP1GAP	2.10973508	0.00019737	CanFam3
RARB	2.0951306	0.00024368	CanFam3
REEP6	2.05807762	0.01324762	CanFam3
RHOU	2.02936712	3.2271E-15	CanFam3
RPTN	2.26218795	0.04615832	CanFam3
SEPTIN12	3.92853849	0.01685146	CanFam3
SGK2	2.21968072	0.00368077	CanFam3
SIRT2	2.06658308	8.7254E-07	CanFam3
SLC16A5	2.35488268	8.6068E-05	CanFam3
SLC18A2	2.68306036	0.00173108	CanFam3
SLC2A5	3.42529927	1.299E-05	CanFam3
SOX10	2.28148074	7.1402E-05	CanFam3
SPATA16	3.01313305	0.00704108	CanFam3
STEAP4	2.19188312	0.01930935	CanFam3
TACR2	2.09095231	0.02089099	CanFam3
TENT5B	2.45632942	1.1123E-05	CanFam3
TLR9	2.95732521	0.00141017	CanFam3
TNFSF13	2.3706556	7.7938E-05	CanFam3
ZC3H12D	2.25338581	0.00205621	CanFam3
ZNF385C	4.57134872	1.4052E-14	CanFam3
ABAT	-2.2599419	1.7601E-05	CanFam4
ABCA8	2.39330882	0.00374339	CanFam4
ABCD2	-4.4932574	7.7201E-05	CanFam4
ADRA1D	2.51122326	5.8707E-06	CanFam4
AFF3	-2.457686	0.00017045	CanFam4
AFMID	-2.9801627	0.00035031	CanFam4
AGAP1	-2.0320764	1.6201E-06	CanFam4
ALPK3	-4.2911276	2.7495E-13	CanFam4
AMIGO1	-2.0834236	9.7062E-06	CanFam4
ANO2	-3.8363156	2.5271E-05	CanFam4
ANXA9	-2.7463587	9.5887E-13	CanFam4
APC2	-2.1825294	0.00096565	CanFam4
AQP12B	-5.3066952	1.2417E-05	CanFam4
AQP3	-3.9909754	4.6978E-05	CanFam4
AQP6	2.73624852	0.0306846	CanFam4
ARHGAP4	-2.171556	2.2308E-07	CanFam4
ARSL	-3.4283244	0.00038531	CanFam4
ASB1	-2.0209048	8.9622E-09	CanFam4
ASB11	-3.8779191	1.0236E-06	CanFam4
ASB5	-3.2799968	0.00760142	CanFam4
ASCL2	2.63254509	0.01578862	CanFam4
ASL	2.40485812	8.3237E-10	CanFam4
ATP1B4	3.94410094	0.00306683	CanFam4
BFSP2	-5.6598113	4.8153E-06	CanFam4
BGLAP	-5.427167	4.3722E-15	CanFam4
BHLHA15	-3.4184204	1.032E-07	CanFam4
BHLHE22	-6.1885305	1.9026E-07	CanFam4
BIK	-3.2537526	9.8947E-06	CanFam4
BLID	-3.3956612	1.6653E-07	CanFam4
BTC	-5.8709851	0.00089392	CanFam4
C17H2orf16	-2.279842	0.00197374	CanFam4
C1H19orf33	3.5235548	6.5094E-08	CanFam4
C1QL3	-4.7816107	3.0612E-08	CanFam4
C1QTNF4	-3.5702016	1.9354E-10	CanFam4
C24H20orf204	-2.6332923	0.00078786	CanFam4
C29H8orf89	2.79804196	4.8771E-07	CanFam4
C2CD4A	-4.1492079	5.1981E-14	CanFam4
C2CD4B	-4.1980303	1.9766E-16	CanFam4
C4H5orf58	-2.9567297	2.5241E-07	CanFam4
C6H1orf194	-2.1132865	2.3522E-05	CanFam4
CABP1	-2.3500891	1.5855E-06	CanFam4
CAMK2N2	-4.6359449	1.7797E-16	CanFam4
CAPN11	2.1485031	0.00698656	CanFam4
CAPN12	2.53950615	2.2826E-07	CanFam4
CARD8	-2.4876268	1.5116E-06	CanFam4
CASQ1	2.20426707	0.0489752	CanFam4
CBS	-3.2049002	0.00016342	CanFam4
CC2D2B	-2.0122288	0.00165032	CanFam4
CCDC169	2.54632047	0.00250622	CanFam4
CCDC180	-2.1260035	0.00287562	CanFam4
CCDC40	-5.8413235	9.1166E-21	CanFam4
CCNJL	3.34740652	8.8148E-18	CanFam4
CD1E	2.6301633	0.00222667	CanFam4
CD24	2.18339141	8.1842E-08	CanFam4
CD247	-2.6839422	4.1972E-07	CanFam4

**CDC42EP4**	2.74227894	6.0051E-07	CanFam4
**CEACAM23**	-2.4475175	0.00432345	CanFam4
**CEL**	-2.3116308	0.00028764	CanFam4
**CELF6**	-6.4898633	4.404E-19	CanFam4
**CES5A**	-5.7563084	5.2527E-13	CanFam4
**CFAP47**	6.43127897	3.206E-12	CanFam4
**CFAP53**	-2.0225312	0.00031589	CanFam4
**CGREF1**	-2.0153425	0.00344439	CanFam4
**CHADL**	2.10679793	0.00063302	CanFam4
**CHGA**	-4.1488236	0.00025203	CanFam4
**CHPT1**	3.34865596	2.1668E-08	CanFam4
**CHRNA3**	-5.7357115	1.5027E-08	CanFam4
**CHST13**	-6.6082511	2.9392E-29	CanFam4
**CLCF1**	2.57351763	0.00065668	CanFam4
**CLCNKA**	-2.6451894	0.01644808	CanFam4
**CLDN23**	2.41239911	0.01037436	CanFam4
**CLDND2**	-2.4062586	0.00049688	CanFam4
**CLIC6**	-8.4183147	4.3383E-22	CanFam4
**CMSS1_1**	3.28309756	1.3493E-09	CanFam4
**CNGA4**	-3.0836306	7.4436E-08	CanFam4
**COL3A1**	-2.0011813	0.01468035	CanFam4
**CTLA4**	-2.088389	0.04652509	CanFam4
**CTSV**	2.32630053	7.7241E-07	CanFam4
**CTXND1**	-7.6123699	2.9387E-16	CanFam4
**CYSRT1**	-2.5016164	0.00201569	CanFam4
**DCX**	-5.6925884	0.0021153	CanFam4
**DEUP1**	-3.7195433	0.00075226	CanFam4
**DHRS2**	-2.5888788	0.02917627	CanFam4
**DIO3**	-3.0198212	0.0015936	CanFam4
**DIPK2A**	2.63363639	0.00044266	CanFam4
**DIRAS1**	-3.1748347	0.00029377	CanFam4
**DLX6**	3.02689032	0.00629912	CanFam4
**DNAJC6**	-2.5621389	8.4595E-06	CanFam4
**DRC1**	3.53104866	7.2656E-05	CanFam4
**DRP2**	-2.9483183	0.00322727	CanFam4
**DSC3**	4.88890696	1.6877E-05	CanFam4
**DTX4**	2.3612198	0.00222091	CanFam4
**EEF1A2**	-2.9609793	0.03601082	CanFam4
**EFNB3**	-3.844732	1.1497E-07	CanFam4
**EFS**	2.11962753	0.0279724	CanFam4
**ELFN1**	-2.414771	0.00955612	CanFam4
**ELOVL7**	3.71047433	6.1924E-14	CanFam4
**ENHO**	-3.2090247	0.00012006	CanFam4
**EPHA6**	-3.4923817	1.3504E-07	CanFam4
**EPHX4**	-3.1530967	0.00095886	CanFam4
**FAM133A**	-3.9962899	0.01461612	CanFam4
**FAM174B**	-3.0734036	4.8771E-07	CanFam4
**FAM181B**	2.74627359	4.9166E-05	CanFam4
**FAM20A**	-2.1438734	4.0169E-05	CanFam4
**FAM25A**	3.83877122	1.6965E-06	CanFam4
**FAM3D**	3.00471634	0.00207055	CanFam4
**FBXO32**	-2.0055163	1.7644E-07	CanFam4
**FEZF1**	3.79272178	0.03883601	CanFam4
**FGF23**	-2.2483732	0.00652912	CanFam4
**FJX1**	-2.5956831	2.565E-06	CanFam4
**FNDC3B**	2.01806245	3.5839E-11	CanFam4
**FOXD1**	2.67598616	0.00755164	CanFam4
**FOXE1**	4.65806995	1.2188E-08	CanFam4
**FOXN4**	-4.8317272	1.7398E-07	CanFam4
**FRMD1**	3.55616709	0.00055728	CanFam4
**FSCN2**	-3.2492881	4.8111E-08	CanFam4
**FUT7**	-2.4029293	1.6877E-05	CanFam4
**GABRR1**	-5.9339316	6.6635E-12	CanFam4
**GALNT16**	-2.6066735	0.002641	CanFam4
**GBX1**	-2.8602348	5.424E-07	CanFam4
**GCNT1**	2.8358525	0.00013887	CanFam4
**GDF6**	-5.1556698	1.487E-12	CanFam4
**GIMAP6**	-5.0208836	1.0883E-25	CanFam4
**GIMAP7**	-2.0453278	0.0001922	CanFam4
**GP9**	2.66682719	1.6965E-06	CanFam4
**GPR135**	-3.7853132	1.9113E-11	CanFam4
**GPR174**	-2.3538657	0.00563181	CanFam4
**GPT2**	2.56900896	4.5721E-08	CanFam4
**GRID2IP**	-2.4631681	9.9548E-09	CanFam4
**GRIN2C**	-3.3500115	0.04358797	CanFam4
**GRIN3B**	-2.6309204	0.00054345	CanFam4
**GZMA**	-2.1920899	0.0229211	CanFam4
**H2BU2**	-3.4938136	0.00403898	CanFam4
**HAPLN4**	-3.4564875	1.2663E-05	CanFam4
**HENMT1**	3.36096195	0.01928025	CanFam4
**HES2**	-6.6829828	5.2955E-17	CanFam4
**HES6**	-2.3592301	0.003835	CanFam4
**HEY2**	2.62656123	0.00127282	CanFam4
**HMGA2**	5.5925974	6.4876E-10	CanFam4
**HOXD8**	-2.333311	1.7112E-05	CanFam4
**HPX**	-7.3455556	3.486E-49	CanFam4
**HS3ST3A1**	3.20725668	0.02322572	CanFam4

**HS3ST6**	-5.8140401	5.2405E-07	CanFam4
**HSD17B3**	2.54740527	0.00329143	CanFam4
**HTR5A**	-3.4614247	0.00941323	CanFam4
**IFGGC1**	-2.4982032	0.00288354	CanFam4
**IGFBP1**	-9.469281	6.6692E-28	CanFam4
**IGFBPL1**	-5.4779215	7.7778E-20	CanFam4
**IGLON5**	-3.9501721	1.9419E-05	CanFam4
**IMPG1**	-2.7484501	0.0033799	CanFam4
**INSM1**	-9.4298531	8.2257E-13	CanFam4
**IPCEF1**	5.30856246	2.5273E-23	CanFam4
**IRF7**	-2.2351802	0.00056602	CanFam4
**IRS1**	2.23860393	2.7009E-05	CanFam4
**ISL2**	4.34550659	0.00041383	CanFam4
**ITIH3**	-2.1396873	0.00142865	CanFam4
**ITIH4**	-2.5874202	0.00272835	CanFam4
**ITPRIPL1**	2.31484924	1.0901E-14	CanFam4
**JAKMIP3**	-6.7643489	3.9045E-20	CanFam4
**KCNH7**	-2.4966637	0.00308481	CanFam4
**KCNJ1**	5.24829591	0.00026937	CanFam4
**KIAA0408**	-4.1218053	0.00033653	CanFam4
**KLRG2**	3.83056991	1.3406E-09	CanFam4
**KRT124**	-2.8552452	0.02330716	CanFam4
**KRT19**	-3.7472865	0.00586623	CanFam4
**KRT5**	-3.1332538	0.00224751	CanFam4
**LCA5L**	-2.1609973	0.00011224	CanFam4
**LCN2**	2.36736046	0.0497675	CanFam4
**LCN8**	-3.6635694	6.5931E-07	CanFam4
**LETM2**	-2.1427875	6.4514E-08	CanFam4
**LINGO3**	3.09274079	0.00086909	CanFam4
**LIPH**	-4.3356672	7.8506E-09	CanFam4
**LONRF2**	-2.0301854	7.6408E-05	CanFam4
**LRATD2**	-2.8386861	5.5698E-07	CanFam4
**LRRC2**	-8.7178768	1.0671E-06	CanFam4
**LRRC26**	-2.4638327	0.00319383	CanFam4
**LURAP1**	-2.1279511	0.01736612	CanFam4
**LYPD1**	-6.2241284	5.6879E-09	CanFam4
**LYPD6B**	2.35208955	0.00393951	CanFam4
**MARCHF4**	-6.846264	2.0105E-25	CanFam4
**MDGA1**	2.12193166	0.0166909	CanFam4
**MICA**	4.1446155	0.00486711	CanFam4
**MIR10A**	-4.0615803	0.00057586	CanFam4
**MIR125B-1**	2.05113042	0.01335264	CanFam4
**MIR135A-2**	4.06248957	0.00135584	CanFam4
**MIR135B**	6.00052763	3.0295E-06	CanFam4
**MIR147**	-2.0577506	0.02162816	CanFam4
**MIR1837-2**	-2.4578731	0.00086272	CanFam4
**MIR190A**	2.68681344	0.00218659	CanFam4
**MIR218-1**	-3.0046316	0.00404558	CanFam4
**MIR221**	2.44756197	0.02646057	CanFam4
**MIR26A-1**	2.77106294	0.00441474	CanFam4
**MIR365-2**	2.04280063	0.00279889	CanFam4
**MIR8795**	-3.0200386	0.00284003	CanFam4
**MIR8810**	-3.8892797	9.6223E-08	CanFam4
**MIR8822**	-2.1614828	4.0969E-08	CanFam4
**MIR8828**	4.35110528	0.00055301	CanFam4
**MIR8851**	4.09903805	0.03182802	CanFam4
**MMD**	2.11140685	0.00084813	CanFam4
**MPV17L**	-6.7014467	1.273E-20	CanFam4
**MT-III**	5.78389035	3.6219E-12	CanFam4
**MT1E**	4.67939427	9.927E-08	CanFam4
**MTNR1A**	5.5147572	0.00548899	CanFam4
**MUC5AC**	-2.2447285	0.01495144	CanFam4
**MX1**	-3.411507	1.6664E-08	CanFam4
**MYH16**	-2.1650098	8.0862E-05	CanFam4
**MYL7**	2.09444513	0.00125491	CanFam4
**MYO15A**	-2.7993023	0.00020165	CanFam4
**NANOS3**	-2.0150086	0.00060575	CanFam4
**NECAB2**	-3.5238086	3.1296E-05	CanFam4
**NEPN**	2.79163985	0.01411854	CanFam4
**NINJ2**	2.01314617	1.192E-07	CanFam4
**NLGN4X**	-3.7077982	0.01355317	CanFam4
**NPAS3**	-2.0745846	0.00503977	CanFam4
**NPFFR1**	-2.9501485	0.00018104	CanFam4
**NPM2**	-5.4610499	2.2119E-22	CanFam4
**NPPC**	-2.4808453	0.04239335	CanFam4
**NR2E3**	-4.7778903	0.00039937	CanFam4
**NTM**	2.98978882	0.01720723	CanFam4
**NTRK2**	-2.3113898	0.04878186	CanFam4
**ODF3L2**	-3.1500427	0.02942761	CanFam4
**ONECUT1**	-2.3638615	0.0124924	CanFam4
**ONECUT2**	-3.611163	0.00013409	CanFam4
**OTC**	-4.3746656	3.2665E-08	CanFam4
**PABPC5**	-2.8756209	2.6629E-08	CanFam4
**PABPN1L**	-2.8921757	4.4562E-06	CanFam4
**PAQR9**	-4.1406711	9.9849E-07	CanFam4
**PARVG**	-2.4646273	6.6635E-12	CanFam4
**PBX4**	2.05539524	8.0859E-08	CanFam4

**PDE4DIPP2**	-2.1057464	1.0919E-05	CanFam4
**PDGFD**	-2.0419423	0.00104852	CanFam4
**PERCC1**	-4.6068255	5.1807E-07	CanFam4
**PIANP**	-2.186928	0.04976049	CanFam4
**PLA2G2C**	4.20173863	6.6531E-07	CanFam4
**PLA2G4E**	-3.5334193	1.5541E-06	CanFam4
**PLAC8B**	-2.7180938	1.7638E-06	CanFam4
**PLEKHF2**	2.35338162	1.3543E-07	CanFam4
**PNMA8A**	-2.3366013	0.01227896	CanFam4
**PNMA8C**	-8.0036686	9.3989E-18	CanFam4
**POU6F2**	2.27201004	0.03654346	CanFam4
**PRKCG**	-3.2691362	8.1802E-08	CanFam4
**PROKR1**	-4.3173132	2.2301E-07	CanFam4
**PRSS50**	3.71186529	0.02984021	CanFam4
**PVALB**	3.14825934	0.02476782	CanFam4
**RARRES2**	-2.004106	5.0179E-05	CanFam4
**RBPMS2**	2.44364206	0.00018462	CanFam4
**RECQL4**	-3.0600347	1.7014E-06	CanFam4
**REN**	2.13698026	0.01012713	CanFam4
**RNF182**	-2.2267649	0.00479222	CanFam4
**RSPH1**	-2.507939	0.00060535	CanFam4
**S100A4**	-2.2672779	0.00083927	CanFam4
**SATL1**	2.06374081	0.00056575	CanFam4
**SAXO1**	3.6603701	0.0210456	CanFam4
**SCGB1C1**	-3.4214915	1.0496E-06	CanFam4
**SCIN_1**	-2.3849128	0.04773675	CanFam4
**SCML4**	-3.0116216	3.8037E-07	CanFam4
**SCRN1**	-2.4344612	0.03396642	CanFam4
**SCRT1**	-7.2046418	6.7486E-43	CanFam4
**SCUBE2**	3.30315509	5.7431E-05	CanFam4
**SDC2**	2.08737608	1.6312E-05	CanFam4
**SDR42E2**	-4.5827476	2.4112E-08	CanFam4
**SERHL2**	-2.0443871	1.639E-05	CanFam4
**SFTPB**	5.45611237	0.00020739	CanFam4
**SH2D1A**	-2.5822405	3.33E-05	CanFam4
**SHC2**	-2.5781128	0.00290947	CanFam4
**SHISA7**	-2.7764476	0.00115282	CanFam4
**SIGLEC11**	-2.0239537	0.00131431	CanFam4
**SLC22A1**	3.21843876	0.00478267	CanFam4
**SLC24A2**	-3.0371986	4.8608E-09	CanFam4
**SLC25A53**	-5.254355	5.8602E-06	CanFam4
**SLC26A9**	2.20500672	0.00559549	CanFam4
**SLC43A2**	-2.0785796	0.00104295	CanFam4
**SMIM43**	3.36603269	0.0019092	CanFam4
**SMPDL3B**	2.77905604	0.00014689	CanFam4
**SNPH**	-2.6649277	0.00012992	CanFam4
**SOGA3**	-4.0437403	0.00037572	CanFam4
**SOWAHA**	-3.197105	0.00423525	CanFam4
**SOX9**	2.72143989	1.7054E-05	CanFam4
**SPATC1**	-2.0972788	0.01488231	CanFam4
**SPIB**	-2.5998955	5.4406E-05	CanFam4
**SRRM4**	-4.1062331	3.8963E-06	CanFam4
**ST6GALNAC3**	2.19892532	0.00021229	CanFam4
**SULT2B1**	-4.0011752	0.00200242	CanFam4
**SYCP3**	2.16156235	0.00496466	CanFam4
**TAMALIN**	-3.4944183	4.7494E-13	CanFam4
**TCEAL3**	-2.2491176	6.6791E-05	CanFam4
**TCEAL6**	-3.6628911	0.00819542	CanFam4
**TEX12**	-2.5347761	0.00112566	CanFam4
**TEX26**	-4.3174768	6.1887E-05	CanFam4
**TEX35**	5.3325548	0.00251665	CanFam4
**TEX47**	2.67172537	0.00176952	CanFam4
**TGM3**	-4.8475274	4.0129E-05	CanFam4
**TLCD3B**	-6.5690543	5.6624E-21	CanFam4
**TLE6**	-2.3213803	0.00051506	CanFam4
**TLR9_2**	3.04023073	0.0001519	CanFam4
**TMEM106A**	2.02698634	0.0065433	CanFam4
**TMEM132D**	-3.89032	2.1751E-06	CanFam4
**TMEM200C**	-4.6694934	3.4285E-08	CanFam4
**TMEM215**	3.33286185	3.9993E-06	CanFam4
**TMEM235**	-2.7522682	0.00434591	CanFam4
**TMEM271**	2.54680135	0.04361782	CanFam4
**TMOD2**	-5.647663	1.7802E-16	CanFam4
**TNR**	-8.8732525	1.3802E-25	CanFam4
**TRNAK-CUU_16**	2.22765259	0.00231227	CanFam4
**TRNAK-CUU_40**	5.14291353	0.00867107	CanFam4
**TRNAK-UUU_22**	-3.9169023	0.00012658	CanFam4
**TRNAQ-UUG_2**	3.62180861	0.03081046	CanFam4
**TRNAR-UCU_3**	3.99355343	0.00310404	CanFam4
**TRPM2**	-3.2015067	9.7473E-10	CanFam4
**TRPV4**	2.41916631	0.0093396	CanFam4
**TSKS**	3.0028098	0.00670141	CanFam4
**TSPAN7**	-4.8110665	4.2618E-29	CanFam4
**TTC6**	-6.0443618	4.1716E-11	CanFam4
**TUBB3**	-3.4114567	2.2549E-08	CanFam4
**UBD**	-3.2747666	0.00058659	CanFam4
**UCP1**	-6.0632409	5.465E-06	CanFam4

**USP46**	2.06183536	0.00188797	CanFam4
**VSNL1**	-7.53891	2.2773E-16	CanFam4
**WDR64**	3.0378836	2.8168E-05	CanFam4
**XKRX**	-3.5302039	3.4591E-05	CanFam4
**ZAP70**	-2.0653397	6.8985E-05	CanFam4
**ZNF300**	-2.3283586	9.4982E-05	CanFam4
**ZNF391**	2.0010845	4.2551E-06	CanFam4
**ZWINT**	2.40426102	0.00052974	CanFam4
