# Supplementary material for: Comparative genomic and clinicopathological analysis uncovers contrasting molecular profiles of canine and human thyroid carcinomas
Source: Commun Biol. 2025 Dec 6;9:4. doi: 10.1038/s42003-025-09225-y (PMC12765017; doi:10.1038/s42003-025-09225-y)
Supplement: Supplementary file 2 — Description of Additional Supplementary Files [file 42003_2025_9225_MOESM2_ESM.pdf]

Description of Additional Supplementary Files

Supplementary Data 1.

Description: Clinicopathologic data for 60 canine thyroid carcinomas with mutational burden for 30 cases. 34 781

Supplementary Data 2.

Description: Spearman correlation coefficients between Thyroid Differentiation 803 Score (TDS) and gene expression levels across full cohort of thyroid tumors.

Supplementary Data 3.

Description: Spearman correlation coefficients between Thyroid Differentiation Score (TDS) and gene expression levels across 25 canine FTC tumors.

Supplementary Data 4.

Description: Pathways or gene sets enriched in MTC and FTC tumors.

Supplementary Data 5.

Description: List of significant DEGs from FTC vs. Normal thyroid comparison (log2 FC>2; FDR<0.05).

Supplementary Data 6.

Description: List of significant DEGs from MTC vs. Normal thyroid comparison (log2 FC>2; FDR<0.05).

Supplementary Data 7.

Description: List of variants identified via WES pipeline.

Supplementary Data 8.

Description: List of variants identified via RNAseq pipeline.

Supplementary Data 9.

Description: List of gene fusions identified via RNAseq pipeline.

Supplementary Software 1.

Description: Cross-species amino acid position mapping: from dog to human
