# Supplementary material for: Comparative genomic and clinicopathological analysis uncovers contrasting molecular profiles of canine and human thyroid carcinomas
Source: Commun Biol. 2025 Dec 6;9:4. doi: 10.1038/s42003-025-09225-y (PMC12765017; doi:10.1038/s42003-025-09225-y)
Supplement: Supplementary file 4 — Supplementary Software 1 [file 42003_2025_9225_MOESM4_ESM.zip › Supplementary_Software_1/README.html]

README


# Cross-Species Amino Acid Position Mapping: From Dog to Human

This archive contains scripts and example data used to map canine
amino acid positions to orthologous human residues using BLASTp and
R.

## 📁 Folder Contents

- **Bash\_GenerateBLAST\_XML\_Database** - Files and
  Scripts to generate BLASTp XML for Steps 1 - 3.
- **R\_Extract\_Homologous\_Human\_AA\_Positions** - Files
  and Scripts to extract orthologous amino acid position from canine
  peptides to human

---

## 📦 Requirements

- **BLAST+** v2.12.0+
- **seqtk** v1.5-r133
- **R** v4.1.2
  - R packages: `XML`, `readr`, `xml2`,
    `rentrez`

---

## ⚙️ Instructions

### Step 1: Extract peptide sequences for genes of interest from both human and canine peptide FASTA files

#### 🔹 Input

- **Protein databases:**
  - `InputFiles/InputProteinSequences/Homo_sapiens.GRCh38.pep.all.fa`
  - `InputFiles/InputProteinSequences/Canis_familiaris.CanFam3.1.pep.all.fa`
- **List of peptide sequence IDs:**
  - `InputFiles/ListOfProteinIDs/Listof_Human_CancerGenesOfInterest_Seqtk_Input.txt`
  - `InputFiles/ListOfProteinIDs/Listof_Canine_CancerGenesOfInterest_Seqtk_Input.txt`

#### 🔹 Bash Script

Run:

```
   `sh Scripts/01.Extracting_Peptide_Sequences.sh`
```

#### 🔹 Output

- `ExtractedProteinSequences/Extracted_Protein_Sequences_Human_GRCH38_CancerGenesofInterest.fasta`
- `ExtractedProteinSequences/Extracted_Protein_Sequences_Canine_CanFam3_CancerGenesofInterest.fasta`

---

### Step 2: Create BLAST database for human peptide sequences

#### 🔹 Input

- `ExtractedProteinSequences/Extracted_Protein_Sequences_Human_GRCH38_CancerGenesofInterest.fasta`

#### 🔹 Bash Script

Run: `sh Scripts/02.MakeblastDB_Human_GRCH38.sh`

#### 🔹 Output

```
- `BLAST_DataBase/HomoSapiens_CG_proteinBlastDB_GRCH38`
```

---

### Step 3: Run blastp

#### 🔹 Input

- `ExtractedProteinSequences/Extracted_Protein_Sequences_Canine_CanFam3_CancerGenesofInterest.fasta`
- `BLAST_DataBase/HomoSapiens_CG_proteinBlastDB_GRCH38`

#### 🔹 Bash Script

Run:

```
  `sh Scripts/03.BlastP_Canine_Cancer_Peptides_Against_HumanBlastDatabase.sh`
```

#### 🔹 Output

```
- `Canine_BLASTP_To_Human_CancerGenes.xml`
```

---

### Step 4: Map canine amino acid positions to human orthologous positions

#### 🔹 Input

- `../../Bash_GenerateBLAST_XML_Database/BLAST_Outputs/Canine_BLASTP_To_Human_CancerGenes.xml`
- `../InputFiles/Canine_ThyroidCarcinoma_Missense_Mutations.csv`

#### 🔹 R Script

Run in R studio:

```
  - Script name: CrossSpecies_AA_PositionMapping.R
```

#### 🔹 Outputs

```
- 01.Thyroid_Carcinoma_Mutect2_CanFam3_Canine2Human_Variant_LiftOver.csv.csv 
       - Contains homologous amino acid positions mapped from canine to human.
       - Includes reformatted input for downstream FATHMM analysis.

- 02.Thyroid_Carcinoma_Mutect2_CanFam3_FATHMM_Cancer_Data_Output.tab
        - Output from FATHMM (Cancer module): https://fathmm.biocompute.org.uk/cancer.html
        - Provides pathogenicity predictions based on human-mapped amino acid positions.

- 03.Thyroid_Carcinoma_Mutect2_CanFam3_FATHMM_DiseaseOntology_Data_Output.tab
        - Output from FATHMM (Inherited disease ontology module): https://fathmm.biocompute.org.uk/inherited.html
        - Provides disease ontology predictions for the mapped variants.

- 04.Thyroid_Carcinoma_Mutect2_CanFam3_FATHMM_Predictions.csv
        - Final merged dataset of canine amino acid variants mapped to homologous human positions, annotated with FATHMM-predicted disease and cancer relevance.
```
