# Supplementary material for: Comparative genomic and clinicopathological analysis uncovers contrasting molecular profiles of canine and human thyroid carcinomas
Source: Commun Biol. 2025 Dec 6;9:4. doi: 10.1038/s42003-025-09225-y (PMC12765017; doi:10.1038/s42003-025-09225-y)
Supplement: Supplementary file 5 — Reporting Summary [file 42003_2025_9225_MOESM5_ESM.pdf]

nature portfolio

Corresponding author(s): Samantha N. Schlemmer

Last updated by author(s): Oct 21, 2025

Reporting Summary

Nature Portfolio wishes to improve the reproducibility of the work that we publish. This form provides structure for consistency and transparency in reporting. For further information on Nature Portfolio policies, see our Editorial Policies and the Editorial Policy Checklist.

Statistics

For all statistical analyses, confirm that the following items are present in the figure legend, table legend, main text, or Methods section.

n/a	Confirmed	
☐	☒	The exact sample size (*n*) for each experimental group/condition, given as a discrete number and unit of measurement
☐	☒	A statement on whether measurements were taken from distinct samples or whether the same sample was measured repeatedly
☐	☒	The statistical test(s) used AND whether they are one- or two-sided<br>*Only common tests should be described solely by name; describe more complex techniques in the Methods section.*
☐	☒	A description of all covariates tested
☐	☒	A description of any assumptions or corrections, such as tests of normality and adjustment for multiple comparisons
☐	☒	A full description of the statistical parameters including central tendency (e.g. means) or other basic estimates (e.g. regression coefficient) AND variation (e.g. standard deviation) or associated estimates of uncertainty (e.g. confidence intervals)
☐	☒	For null hypothesis testing, the test statistic (e.g. *F*, *t*, *r*) with confidence intervals, effect sizes, degrees of freedom and *P* value noted<br>*Give P values as exact values whenever suitable.*
☒	☐	For Bayesian analysis, information on the choice of priors and Markov chain Monte Carlo settings
☒	☐	For hierarchical and complex designs, identification of the appropriate level for tests and full reporting of outcomes
☒	☐	Estimates of effect sizes (e.g. Cohen's *d*, Pearson's *r*), indicating how they were calculated

Our web collection on statistics for biologists contains articles on many of the points above.

Software and code

Policy information about availability of computer code

Data collection	All data collection was based on Illumina sequencing of DNA and RNA libraries from canine thyroid tumor and matched normal tissues. In addition, we downloaded RNAseq fastq files of normal thyroid tissues from an open-access dog epigenomic resource, BarkBase (https://barkbase.org/, BioProject PRJNA396033).
Data analysis	The Bioinformatic pipelines used in this study can be accessed here: https://github.com/sdas1425/Canine_ThyroidCarcinoma_Omics_Pipeline.git.<br>In addition, we have provided all the custom codes, input and output files to replicate amino acid position mapping method (Supplementary Software 1).

For manuscripts utilizing custom algorithms or software that are central to the research but not yet described in published literature, software must be made available to editors and reviewers. We strongly encourage code deposition in a community repository (e.g. GitHub). See the Nature Portfolio guidelines for submitting code & software for further information.

Data

Policy information about availability of data

All manuscripts must include a data availability statement. This statement should provide the following information, where applicable:

- Accession codes, unique identifiers, or web links for publicly available datasets
- A description of any restrictions on data availability
- For clinical datasets or third party data, please ensure that the statement adheres to our policy

The raw fastq files for WES have been submitted to SRA database under BioProject number: PRJNA1222422. The RNAseq fastq files have been submitted to GEO database under accession number: GSE289443.

Research involving human participants, their data, or biological material

Policy information about studies with human participants or human data. See also policy information about sex, gender (identity/presentation), and sexual orientation and race, ethnicity and racism.

Reporting on sex and gender	N/A
Reporting on race, ethnicity, or other socially relevant groupings	N/A
Population characteristics	N/A
Recruitment	N/A
Ethics oversight	N/A

Note that full information on the approval of the study protocol must also be provided in the manuscript.

Field-specific reporting

Please select the one below that is the best fit for your research. If you are not sure, read the appropriate sections before making your selection.

☒ Life sciences ☐ Behavioural & social sciences ☐ Ecological, evolutionary & environmental sciences

For a reference copy of the document with all sections, see nature.com/documents/nr-reporting-summary-flat.pdf

Life sciences study design

All studies must disclose on these points even when the disclosure is negative.

Sample size	The cohort was determined based on cases submitted to the CSU FACC Biorepository by the time of study initiation (2019).
Data exclusions	Dogs with metastasis at time of diagnosis were excluded from progression free interval analysis because they had gross spread of disease and would alter the interval from dogs without disease spread.
Replication	Singular specimens were available for initial analysis and all material was used, prohibiting replication.
Randomization	Dogs were included in the study if they had naturally-occurring thyroid carcinoma and underwent surgical excision with specimen collection for the CSU FACC Biorepository by the time of study initiation (2019).
Blinding	Pathologists (SNS, DPR) were blinded to patient information and sequencing results.

Reporting for specific materials, systems and methods

We require information from authors about some types of materials, experimental systems and methods used in many studies. Here, indicate whether each material, system or method listed is relevant to your study. If you are not sure if a list item applies to your research, read the appropriate section before selecting a response.

Materials & experimental systems

n/a	Involved in the study	
☐	☒	Antibodies
☒	☐	Eukaryotic cell lines
☒	☐	Palaeontology and archaeology
☐	☒	Animals and other organisms
☒	☐	Clinical data
☒	☐	Dual use research of concern
☒	☐	Plants

Methods

n/a	Involved in the study	
☒	☐	ChIP-seq
☒	☐	Flow cytometry
☒	☐	MRI-based neuroimaging

Antibodies

Antibodies used	Calcitonin (Abcam, ab8553; 1:200 dilution)<br>HER2 (Dako, A0485; 1:400 dilution)
Validation	Calcitonin: validated based on immunoreactivity in canine thyroid (normal and carcinoma) tissue specimens - personal communication with Kansas State Veterinary Diagnostic Laboratory<br>HER2: previously validated on canine cells/tissue (https://doi.org/10.1177/0300985818817024)

Animals and other research organisms

Policy information about studies involving animals; ARRIVE guidelines recommended for reporting animal research, and Sex and Gender in Research

Laboratory animals	N/A
Wild animals	N/A
Reporting on sex	Sex of dogs was captured in the clinicopathologic data and included in pathological, sequencing, and survival analyses.
Field-collected samples	N/A
Ethics oversight	Dogs were pets and resided with their owners. Their tumor samples were archived during clinical treatment by the Flint Animal Cancer Center Biorepository with owner consent, and the Colorado State University Institutional Animal Care and Use Committee protocol (KP 652) approved the study.

Note that full information on the approval of the study protocol must also be provided in the manuscript.

Plants

Seed stocks	N/A
Novel plant genotypes	N/A
Authentication	N/A
